# Classification of protein quaternary structure by functional domain composition

**DOI:** 10.1186/1471-2105-7-187

**Published:** 2006-04-04

**Authors:** Xiaojing Yu, Chuan Wang, Yixue Li

**Affiliations:** 1 Bioinformatics Center, Shanghai Institutes for Biological Sciences, Chinese Academy of Sciences, 320 Yueyang Road, Shanghai 200031, China; 2 Graduate School of the Chinese Academy of Sciences, 19 Yuquan Road, Beijing 100039, China; 3 Shanghai Center for Bioinformation Technology, 100 Qinzhou Road, 200235 Shanghai, China

## Abstract

**Background:**

The number and the arrangement of subunits that form a protein are referred to as quaternary structure. Quaternary structure is an important protein attribute that is closely related to its function. Proteins with quaternary structure are called oligomeric proteins. Oligomeric proteins are involved in various biological processes, such as metabolism, signal transduction, and chromosome replication. Thus, it is highly desirable to develop some computational methods to automatically classify the quaternary structure of proteins from their sequences.

**Results:**

To explore this problem, we adopted an approach based on the functional domain composition of proteins. Every protein was represented by a vector calculated from the domains in the PFAM database. The nearest neighbor algorithm (NNA) was used for classifying the quaternary structure of proteins from this information. The jackknife cross-validation test was performed on the non-redundant protein dataset in which the sequence identity was less than 25%. The overall success rate obtained is 75.17%. Additionally, to demonstrate the effectiveness of this method, we predicted the proteins in an independent dataset and achieved an overall success rate of 84.11%

**Conclusion:**

Compared with the amino acid composition method and Blast, the results indicate that the domain composition approach may be a more effective and promising high-throughput method in dealing with this complicated problem in bioinformatics.

## Background

The structure hierarchy of proteins is defined in terms of four levels: primary, secondary, tertiary, and quaternary. The term *quaternary structure *was first introduced by Bernal in 1958 [1-3]. It refers to the non-covalent interactions of protein subunits to form oligomers and the spatial arrangement of the subunits.

Oligomeric proteins are very common in nature. They can be divided further into two classes: homo-oligomers and hetero-oligomers; the former are composed of identical subunits while the latter are composed of non-identical subunits. For example, the potassium channel is formed by a homo-tetramer [4] , and the gamma-aminobytyric acid type A (GABA_A_) receptor is formed by a hetero-pentamer [5]. The subunit construction of proteins provides the structural basis for their activities and functions in various biological processes, which include metabolism, signal transduction and chromosome replication [3,6]. From an evolutional point of view, the oligomeric proteins have more advantages than the monomers [7,8]. It is easier for multi-subunit proteins to repair their defects by simply replacing the flawed subunit [9]. Moreover, in a number of biological processes, the quaternary structure of proteins is indispensable for their function [9]. Thus, the study of the quaternary structure is an interesting field in bioinformatics.

It is generally accepted that the amino acid sequence of most proteins contains all the information needed to fold the protein into its correct three-dimensional structure [3,10-12]. The quaternary structure of proteins, which is the association of tertiary structure subunits, depends on the existence of complementary "patches" on their surfaces [12]. Therefore, the patches that are buried in the interfaces formed by the subunits play a vital role in both tertiary and quaternary structures. This suggests the possibility to predict the quaternary structure from primary sequences [12].

The actual quaternary structure features of proteins must be determined by experiments, which are slow and expensive. However, computational methods like machine learning, can extract some valuable information such as the number of subunits from protein amino acid sequences. They may play a role in the study of this issue, when the genome-sequencing project produces such large amounts of sequence information. Some efforts have been made in developing computational tools to predict protein quaternary structure from its sequence. Among them, the methods employed were the decision-tree method with the feature extraction function (the simple binning function) [12] , the support vector machine (SVM) and the covariant discriminant algorithm with two protein sequence descriptors [3] , the pseudo amino acid composition method [9] , and the function of degree of disagreement (FDOD) method [13].

In this paper, the functional domain composition of proteins was initially adopted to investigate the problem. In some previous work, the functional domain information has been used to predict protein-protein interaction [14,15] , protein structure [16] and protein function [17,18] etc. The promising results have indicated that the domain composition of a protein is closely linked with its function and interactions with other proteins. The quaternary structure is closely related to the interactions between the subunits of an oligomer; thus, it's closely related to the functional domains of a protein. Consequently, we chose the functional domain composition as the feature to represent a protein. The present study is limited to homo-oligomers. The jackknife cross-validation test was performed on the protein dataset in which the sequence identity was less than 25%. The overall success rate is 75.17%. In the same dataset, the amino acid composition method and Blast [19] achieved the accuracy of 41.42% and 69.60% respectively. The results demonstrate that the functional domain composition approach is a promising high-throughput method in dealing with this complicated problem in bioinformatics.

## Results and discussion

The computations were carried out on a Dell OptiPlex GX260 computer with an Intel Pentium4 2.40 GHz CPU. It is well known that in statistical prediction, the single independent dataset test, the self-consistency test, and the jackknife test are the three methods often used in algorithm assessment. Among them, the jackknife test is considered the most objective and rigorous way to do cross-validation [20,21]. The success prediction rate in practical application should be measured by the result of the jackknife test, rather than the sub-sampling test or the limited independent dataset test [22,23]. Therefore, in this work, the results acquired from the jackknife test were considered to be the success rates of the functional domain composition approach proposed here.

Table 1 shows the success rates obtained by the domain composition method, the amino acid composition method and Blast in the seven quaternary categories. Every protein in the non-redundant training dataset was predicted by the nearest neighbor algorithm. The overall success rate achieved by the domain composition method is 75.17%. The results indicate that domain composition is a very effective feature of proteins for quaternary structure prediction. In order to demonstrate the effectiveness of the domain composition method, a direct comparison was made between the domain composition method and the sequence amino acid composition method, which is also a frequently used approach in protein sequence analysis [24-27]. The vectors calculated from the sequence amino acid composition in the same dataset were used as the input for NNA. As shown in Table 1, the domain composition method greatly outperformed the sequence amino acid composition method. Moreover, we conducted the jackknife test in the same dataset by Blast [19]. In Blast, we chose the category with the best hit of a query protein as the predicted category of that protein. The corresponding overall rate obtained by Blast is 69.60%, which is about 5.57% lower than the success rate obtained by the domain composition approach (Table 1).

In addition to the jackknife test performed on the training dataset, we predicted all the 9951 proteins in the independent dataset with NNA as well. Each protein in the independent dataset was assigned into the structural category to which its nearest neighbor protein in the non-redundant training dataset belongs. As shown in Table 2, 8370 proteins were correctly classified and the overall accuracy is 84.11%.

Furthermore, we also tried to compare the results with previous studies. Garian employed the decision tree and binning function to build models for classifying homo-dimers from other homo-oligomers, and obtained an accuracy of 69.9% [12]. Zhang et al. used the same dataset to classify homo-dimers by the SVMs and the covariant discriminant algorithms. They obtained overall accuracies ranging from 78.5% to 87.5% by the SVMs and from 58.9% to 79.7% by the covariant discriminant algorithms [3]. Through a tentative comparison in the category of homo-dimers, the results show that we achieved similar or better levels of prediction in terms of accuracy.

## Conclusion

The functional domain composition method is an effective method that has been widely used in protein function prediction [17,28]. In this paper, it illustrates its power in the multi-class prediction of the protein quaternary structure. If we suppose that the protein samples were distributed according to the sizes of categories [9] , then the rate of correct prediction by the measured random assignment would be (208/717)^2 ^+ (335/717)^2 ^+ (40/717)^2 ^+ (95/717)^2 ^+ (11/717)^2 ^+ (23/717)^2 ^+ (5/717)^2^≈ 32.44%. Evidently, the rates of correct prediction acquired by the functional domain composition approach are much higher than the random assignment, which suggests that the quaternary structure of an oligomeric protein can be inferred from its sequence and the function domain composition is a potent feature for quaternary structure prediction. Presently, the quaternary classifier constructed in this paper is limited to homo-oligomers. With the accumulation of experimental data, the future work of quaternary structure prediction will take place in the area of investigating classifiers for hetero-oligomers.

## Methods

### Data sets

We extracted the subunit comment for every entry in the Swiss-Prot database (version 45.4) [29,30] and then used "Monomer", "Homodimer", "Homotrimer", "Homotetramer", "Homopentamer", "Homohexamer", "Homoheptamer", and "Homooctamer" as keywords to search for the oligomeric proteins of each category. Thus, 16819 entries were retrieved. Because there was only one protein in the "Homoheptamer" class, it was removed. Therefore, there were 16818 proteins in the whole dataset. The protein sequences that contain irregular amino acid characters such as "x" and "z" or with a length over 6000aa or less than 50aa were removed. Moreover, redundant sequences in the whole datasets were removed by the CD-HIT [31] and PISCES [32] program, with a threshold of 25%. Altogether, we came up with 1665 proteins in total. However, in the dataset of 1665 proteins, 948 proteins were not suitable for the functional domain composition feature extraction method, because they either could not get hits in the PFAM database [33] or belonged to different classes with exactly the same domain composition. Moreover, some proteins were "orphan proteins", which means none of the domains they contained were shared by other proteins in the dataset. Consequently, the non-redundant training dataset was composed of 717 proteins by further removing those 948 proteins (Table 1). Additionally, in order to test the effectiveness of the domain composition method, we constructed an independent testing dataset. All the proteins that contain the domains involved in the training dataset but are not in it were extracted from the whole dataset. Thus, we obtained the independent testing dataset of 9951 proteins (Table 2). All the data are available in the additional files.

### Functional domain composition feature vector

The use of the functional domain composition to represent a protein was motivated by a series of previous studies of proteins [17,18,28]. Here, the functional domain is defined in the PFAM database, which contains a large collection of multiple sequence alignments and hidden Markov model (HMM) profiles covering many common protein domains and families [33]. The determination of domain boundaries, family members and alignments is performed semi-automatically based on expert knowledge, sequence similarity, HMM-profiles and other protein family databases [34,35]. There are accession number links to the PFAM database in the Swiss-Prot database [30]. Therefore, we searched the PFAM domain annotation in the Swiss-Prot database for these 717 proteins, and recorded all types of domains they contained. The results showed that they totally consisted of 540 types of domains. Thus, the functional domain composition of a protein can be defined as a 540D (dimensional) vector.

For a given protein, if it contains the 11^th ^domain in the recorded domain list, the 11^th ^component of the protein in the 540D functional domain space is assigned 1; otherwise, 0 [16,28]. The protein can thus be explicitly formulated as

X=[x1x2⋮xj⋮x540],
 MathType@MTEF@5@5@+=feaafiart1ev1aaatCvAUfeBSjuyZL2yd9gzLbvyNv2CaerbwvMCKfMBHbqedmvETj2BSbqee0evGueE0jxyaibaieYdOi=BH8vipeYdI8qiW7rqqrFfpeea0xe9Lq=Jc9vqaqpepm0xbbG8FasPYRqj0=yi0lXdbba9pGe9qqFf0dXdHuk9fr=xfr=xfrpiWZqaaeaabiGaaiaacaqabeaabeqacmaaaOqaaiaadIfacqGH9aqpdaWadaqaauaabeGageaaaaqaaiaadIhadaWgaaWcbaGaaGymaaqabaaakeaacaWG4bWaaSbaaSqaaiaaikdaaeqaaaGcbaGaeSO7I0eabaGaamiEamaaBaaaleaacaWGQbaabeaaaOqaaiabl6UinbqaaiaadIhadaWgaaWcbaGaaGynaiaaisdacaaIWaaabeaaaaaakiaawUfacaGLDbaacaGGSaaaaa@41A2@

where xj={1hit,0otherwise.
 MathType@MTEF@5@5@+=feaafiart1ev1aaatCvAUfKttLearuWrP9MDH5MBPbIqV92AaeXatLxBI9gBaebbnrfifHhDYfgasaacH8akY=wiFfYdH8Gipec8Eeeu0xXdbba9frFj0=OqFfea0dXdd9vqai=hGuQ8kuc9pgc9s8qqaq=dirpe0xb9q8qiLsFr0=vr0=vr0dc8meaabaqaciaacaGaaeqabaqabeGadaaakeaacqWG4baEdaWgaaWcbaGaemOAaOgabeaakiabg2da9maaceaabaqbaeqabiGaaaqaaiabigdaXaqaaiabdIgaOjabdMgaPjabdsha0jabcYcaSaqaaiabicdaWaqaaiabd+gaVjabdsha0jabdIgaOjabdwgaLjabdkhaYjabdEha3jabdMgaPjabdohaZjabdwgaLjabc6caUaaaaiaawUhaaaaa@4634@

Consequently, using each of the 540 functional domains as a base, a protein is represented by a 540D vector.

### The Nearest Neighbor Algorithm

The Nearest Neighbor Algorithm (NNA) compares the features of the unknown new samples with the features of the samples that have already been classified, and then, classifies the new samples into their class membership [36,37]. The decision rule of NNA assigns the category of the nearest one of a set of previously classified samples to an unclassified sample. If the distributions and the categories of the samples are unknown, NNA is particularly useful. NNA is easy to implement and has a low error probability [17]. Thus, it is an attractive method to be employed in the bioinformatics study [16,17,20,38].

Suppose that we are given *n *proteins *(x_1_, x_2_, ..., x_n_)*, which have been classified into *m *categories *(c_1_, c_2_, ..., c_m_)*. Then, the category to which an unknown protein *x *belongs can be predicted by the following NNA principle. First, *the generalized distance *between *x *and *x_i_(i = 1, 2, ..., n) *is defined as:

D(x,xi)=1−x⋅xi‖x‖‖xi‖ (i=1,2,...,n),
 MathType@MTEF@5@5@+=feaafiart1ev1aaatCvAUfKttLearuWrP9MDH5MBPbIqV92AaeXatLxBI9gBaebbnrfifHhDYfgasaacH8akY=wiFfYdH8Gipec8Eeeu0xXdbba9frFj0=OqFfea0dXdd9vqai=hGuQ8kuc9pgc9s8qqaq=dirpe0xb9q8qiLsFr0=vr0=vr0dc8meaabaqaciaacaGaaeqabaqabeGadaaakeaacqWGebarcqGGOaakcqWG4baEcqGGSaalcqWG4baEdaWgaaWcbaGaemyAaKgabeaakiabcMcaPiabg2da9iabigdaXiabgkHiTmaalaaabaGaemiEaGNaeyyXICTaemiEaG3aaSbaaSqaaiabdMgaPbqabaaakeaadaqbdaqaaiabdIha4bGaayzcSlaawQa7amaafmaabaGaemiEaG3aaSbaaSqaaiabdMgaPbqabaaakiaawMa7caGLkWoaaaGaeeiiaaccbaGae8hkaGIaemyAaKMaeyypa0JaeGymaeJaeiilaWIaeGOmaiJaeiilaWIaeiOla4IaeiOla4IaeiOla4IaeiilaWIaemOBa4Mae8xkaKccbiGae4hlaWcaaa@57BA@

where *x·x_i _*is the dot product of vectors *x *and *x_i_*. || *x *|| and || *x_i _*|| are their moduli.

When *x ≡ x_i_*, *D*(*x, x_i_*) = 0. In brief, the generalized distance is within the range of 0 and 1; i.e., *D*(*x, x_i_*) ∈ [0,1].

Then, the nearest neighbor of *x *can be defined as *x_k_*,

where

D(x,xk)=min⁡i=1nD(x,xi).
 MathType@MTEF@5@5@+=feaafiart1ev1aaatCvAUfKttLearuWrP9MDH5MBPbIqV92AaeXatLxBI9gBaebbnrfifHhDYfgasaacH8akY=wiFfYdH8Gipec8Eeeu0xXdbba9frFj0=OqFfea0dXdd9vqai=hGuQ8kuc9pgc9s8qqaq=dirpe0xb9q8qiLsFr0=vr0=vr0dc8meaabaqaciaacaGaaeqabaqabeGadaaakeaacqWGebarcqGGOaakcqWG4baEcqGGSaalcqWG4baEdaWgaaWcbaGaem4AaSgabeaakiabcMcaPiabg2da9maaxadabaGagiyBa0MaeiyAaKMaeiOBa4galeaacqWGPbqAcqGH9aqpcqaIXaqmaeaacqWGUbGBaaGccqWGebarcqGGOaakcqWG4baEcqGGSaalcqWG4baEdaWgaaWcbaGaemyAaKgabeaakiabcMcaPiabc6caUaaa@4820@

According to the NNA rule, the query protein *x *is predicted as belonging to the category *c_j _*∈{*c_1_, c_2_,..., c_m_*} if its nearest neighbor *x_k _*belongs to the category *c_j _*∈{*c_1_, c_2_,..., c_m_*}.

The proteins in the training dataset and the independent testing dataset were all defined in the 540D functional domain composition, and then the NNA prediction was carried out based on the proteins in the training dataset.

## Authors' contributions

XJY developed and implemented the algorithm, prepared the datasets, and drafted the manuscript. CW provided assistance in the acquisition of data and carried out the Blast analysis. YXL directed the whole research and critically revised the manuscript. All authors read and approved the final manuscript.

## Supplementary Material

Additional File 1 Swiss-Prot accession number of 717 proteins in the non-redundant training dataset Click here for file

Additional File 2 Swiss-Prot accession number of 9951 proteins in the independent testing dataset Click here for file

## Figures and Tables

**Table 1:** Comparison of overall success rates obtained by the domain composition method, the amino acid composition method, and Blast in the non-redundant training dataset with a sequence identity less than 25%

Quaternary Structure Category	% Accuracy					
	
	Domain composition method		Amino Acid composition method		Blast	
	
	Number correct/total	% Accuracy	Number correct/total	% Accuracy	Number correct/total	% Accuracy
Monomer	169/208	81.25	79/208	37.98	150/208	72.12
Homodimer	269/335	80.30	180/335	53.73	253/335	75.52
Homotrimer	29/40	72.50	12/40	30	27/40	67.50
Homotetramer	52/95	54.74	19/95	20	50/95	52.63
Homopentamer	11/11	100.00	6/11	54.55	11/11	100.00
Homohexamer	7/23	30.43	1/23	4.35	6/23	26.09
Homooctamer	2/5	40.00	0/5	0	2/5	40.00
Total	539/717	75.17	297/717	41.42	499/717	69.60

**Table 2:** Success prediction rates achieved by the domain composition method in the independent testing dataset

Quaternary Structure Category	Number correct/total	% Accuracy
Monomer	2195/2516	87.24
Homodimer	4227/5061	83.52
Homotrimer	354/399	88.72
Homotetramer	1282/1544	83.03
Homopentamer	12/38	31.58
Homohexamer	224/277	80.87
Homooctamer	76/116	65.52
Total	8370/9951	84.11
