# Supplementary material for: Classification of protein quaternary structure by functional domain composition
Source: BMC Bioinformatics. 2006 Apr 4;7:187. doi: 10.1186/1471-2105-7-187 (PMC1450311; doi:10.1186/1471-2105-7-187)
Supplement: Additional File 1 — Swiss-Prot accession number of 717 proteins in the non-redundant training dataset [file 1471-2105-7-187-S1.pdf]

Swissprot Accession Number of 208 Monomers

Q9K9D5 P06547 P20425 Q48656 Q9T0N8 Q8ZGM2 Q07762 O18739 P45802 Q52675
P16276 Q01470 Q9I5W0 Q9ER72 P43827 Q87A20 P48147 Q8Y2D9 Q43007 Q82JF0
P10746 O88202 Q8N6G6 P06776 P55825 P18102 Q91431 Q41258 P45543 P30269
P36177 P39790 P33401 P47356 Q9M9K1 P17573 P33216 Q9PKR0 Q6H9K0 P21529
P19966 Q10159 P72854 P52320 P41496 P04395 Q8FL98 Q8TKG2 P50747 O06220
Q12546 Q13231 Q88BD4 P62631 P29166 O15886 Q7VQX7 P46198 Q9AK82 P09030
P38939 O67411 P25938 P80356 Q9PPZ6 P78024 P51529 O58530 Q40577 P00811
P09126 Q7MXD5 P41915 P06709 P37060 Q8G1N5 P77901 O89107 Q9UI17 P22362
P40114 O68925 P47787 P68641 Q8XYN6 P18330 P81186 P08373 Q9C469 P16456
P14375 P36683 P23354 P27543 P23262 Q56691 P21248 P95339 P00259 Q59288
P19179 O86956 Q7NDF6 Q89WF2 P75426 P57023 P27652 Q978S3 Q03460 P01583
Q29146 P52664 P34024 P55263 O08451 P53354 Q9A919 P83578 O32449 P11916
P20485 Q05884 P31071 P83434 O62650 Q01584 P12676 P45270 O25474 P56926
P55100 P00751 P06530 P18055 Q04656 O32611 Q26643 Q86WA6 P16113 Q9P376
P83913 O04847 P25718 P07998 Q93VA3 Q55012 P14898 P59807 Q07283 Q9KX73
O62554 P23489 Q59752 P58598 Q06846 P22963 P23882 P20160 P17405 P39662
O09175 P42220 Q92H06 Q8XLP3 P22414 P36911 P14550 P33244 P17409 P25997
P01088 P20625 P15888 P25500 P06279 P29717 P29027 P09978 Q59634 Q9PQ53
P46541 Q03603 Q60575 P36913 Q8XWY0 P30924 P29768 O83466 P78423 O14874
P19531 O51768 P05101 Q09629 P63032 P02788 O75688 Q44118 P21499 P28861
Q9Q8L4 Q9BXP8 Q09923 P52718 P01584 O14156 Q9X7R6 P14250

Swissprot Accession Number of 335 Homodimers

Q02989 P27830 P72181 P13466 P17493 Q9X5X4 P45799 Q9X2W0 O83618 O34777
Q7N6I1 P09390 Q07536 P22413 P04043 Q46822 P53164 Q41342 P22106 Q58786
Q9JJL8 Q8DKE4 P78330 O95390 P57416 O70423 P16930 P21632 P30568 O42131
P49767 Q98B00 P52307 P17559 P32754 P04694 P40582 P33330 Q88YC0 P19938
Q99K67 Q60660 P30014 P59926 P39905 P08619 P48496 P54321 P31166 P40817
P26446 P38681 P19881 O13046 Q7V3Q0 P00550 P40200 O69683 Q8L633 Q920H1
P25976 P47364 Q976K1 Q83FF5 O83776 P04169 Q06881 Q9PQ83 P38487 O06899
Q88V56 P32178 P07277 Q9HP68 P83453 Q03154 P11024 P92943 P13377 Q9Z4J7
Q9RUF3 Q08415 P80401 P54071 Q9S3Q2 P41043 Q29451 P04802 Q9Y9D9 O04985
P80860 P32184 P09053 P68579 P54274 P33197 P47990 P13663 P21549 Q04945
O00322 Q8RXR2 O62771 P02224 P01034 Q83GI1 O83678 P06621 Q7NBE4 O83195
P42517 Q9Y946 P46908 P82371 Q7VG78 P35482 Q9ZDL2 P01267 P82957 P32054
P05725 P21631 P63590 P00152 P05340 P16670 P77368 Q03520 Q8CP16 O96553
Q02046 Q8TXX9 P31896 P21266 Q9UK05 P41142 P14740 P20115 P31441 P20135
O51418 P01398 Q83FI4 Q97BQ3 Q801X7 P33023 P12996 P27046 P28305 P34736
P05057 Q62967 P50384 Q9Z5X5 P16574 Q9KJU0 P43026 Q16222 Q9R8E3 P52960
P37217 P19971 Q05597 Q9YC66 P15692 P03951 P47525 P08836 P30821 P30038
P94135 O00408 Q89RW1 Q03042 O85341 P04907 Q6D5V7 Q9Z7U6 P35146 P32921

P29079 P26639 P80748 P22984 Q04515 P31654 Q57997 P72292 P12045 P28821
P46883 Q9CMY2 P12955 Q55653 P13195 Q96291 P00210 Q8GB19 Q9VG93 Q27783
O84339 P31660 Q8PZI1 P17342 P14748 P97544 Q02457 Q8J0N6 P00154 Q8UI98
P83618 O86109 Q9RUH3 P16108 Q00955 P02212 Q8FM79 Q7U3K6 Q91Y57 P26976
Q8NJR4 P08813 Q8D2K6 P25848 P21854 Q8ZA85 P27995 P07445 Q55393 Q9UKK9
P54787 P00260 P23385 Q05733 O00519 P63500 Q6F6Z6 O74928 P03878 Q9LQ10
Q08420 P28676 Q07257 Q58083 P33186 P58497 Q81FS9 O95848 P49025 P59304
P54690 Q9PMN3 Q9R111 P44521 P46450 P15180 Q92496 P80324 Q9F0J6 Q9JJH5
P63498 Q02959 P21639 P58350 P41399 O31817 Q10039 Q9S7B5 P07642 Q968X7
P34913 P29473 O08404 Q10711 Q12882 P14805 O42281 P19368 P77407 P17423
O94833 Q6L0S8 O42242 O51160 P33764 P03972 Q7ZZC8 Q89AS0 O44476 P15309
Q9PIC1 Q7VZ05 Q7NAN6 P09367 Q53464 Q03298 P34755 Q8CPS8 O67115 O86447
Q59106 P41148 Q9A6T4 Q9YGP1 Q9NSU2 Q59452 P61444 P78417 Q9UP52 P54697
P26563 P95333 Q7VIJ3 P08466 P03817 P54638 Q8ZA49 Q6KIH8 P74782 P13006
P57221 P63201 P00144 P15245 P52893 Q9A810 P07738 Q9DGI0 P50442 P37551
P09378 O26255 Q56694 P29219 P47950

Swissprot Accession Number of 40 Homotrimers

P37889 Q83457 Q8ZYL6 Q9Z2P3 O43895 P24305 O84441 Q04830 P78605 Q56110
P97085 O75888 P57041 Q02388 Q03553 P32370 P41153 P22813 P21826 P07811
P15873 P41274 Q9I8D8 O55237 Q8JFG3 P22230 Q8G5L2 Q01537 Q9HN45 P19721
O84536 P36844 P32972 Q9YEZ5 P35440 P05055 P56220 P10961 Q9ESE2 P10620

Swissprot Accession Number of 95 Homotetramers

Q8DPZ9 P13702 Q9WYH8 P35790 Q9CYR0 Q971T6 P58839 P07954 Q9QXE0 P81650
P42206 O69782 O76464 O34153 P44527 O18835 P13203 P73534 P65167 Q9UUS2
Q9D684 Q27893 Q24498 Q9YA75 P22498 P37062 Q16698 P44777 Q980W9 P43061
P27867 O66883 P37354 Q7V121 Q83Q93 Q9RFM4 Q8ZB04 P00886 P31218 Q14314
P11274 Q9BYV1 Q88S52 P03023 Q42736 Q06210 O52354 O68897 Q7TVV6 Q50744
Q64467 Q9HSN7 P06613 P15244 Q07982 Q9RA05 P20906 P35433 P00386 P37079
P53780 Q9X0L4 P14941 P52784 Q9CBW3 Q12791 P53529 O26061 Q9YAX2 P00561
P37063 P13676 O50083 P32445 P25306 P29995 P80030 P22178 P33327 Q06528
P28269 P58814 P07246 P56653 P21673 Q9UUZ4 P32232 P29147 Q84H44 P47199
P08576 P13087 P76129 O08651 P29038

Swissprot Accession Number of 11 Homopentamers

P50861 O67805 Q9K6W0 O83842 Q56827 Q56705 Q03475 Q9R9R6 P96786 O51173
P61711

Swissprot Accession Number of 23 Homohexamers

P52158 Q8YN70 P94164 P11436 Q7N8S4 Q57872 P16006 Q8EUG2 P37051 O34714 P52671 P49172 P07374 P37981 P15623 O26253 Q91437 P36775 Q13011 P37066 P54385 P52157 P22143

Swissprot Accession Number of 5 Homooctamers

O30807 P80449 P13564 P31055 P06217
