# Supplementary material for: Classification of protein quaternary structure by functional domain composition
Source: BMC Bioinformatics. 2006 Apr 4;7:187. doi: 10.1186/1471-2105-7-187 (PMC1450311; doi:10.1186/1471-2105-7-187)
Supplement: Additional File 2 — Swiss-Prot accession number of 9951 proteins in the independent testing dataset [file 1471-2105-7-187-S2.pdf]

Swissprot Accession Number of 2516 Monomers

O00748 O02785 O04499 O04939 O05137 O05338 O05593 O06851 O07045 O08498
O08557 O08782 O13966 O14295 O14977 O15182 O16845 O18344 O18345 O18408
O18420 O18552 O18842 O19113 O19906 O24243 O24246 O24706 O25360 O27194
O27428 O29238 O29581 O30520 O30881 O31153 O31297 O31298 O31300 O31301
O32913 O33007 O33120 O33470 O33567 O33594 O33843 O34204 O34403 O34530
O35071 O35188 O35484 O35648 O42207 O42446 O43896 O46612 O46613 O48668
O48882 O50048 O50293 O50306 O50340 O50606 O51126 O51208 O51345 O51378
O51540 O51545 O51653 O51680 O51773 O51934 O52200 O52352 O52720 O52727
O52914 O53084 O53241 O53796 O54326 O54974 O55028 O55145 O57214 O58362
O58792 O58844 O60930 O62829 O62830 O66429 O66490 O66555 O66651 O66893
O66936 O67037 O67068 O67163 O67226 O67271 O67298 O67465 O67494 O67653
O68142 O68844 O68901 O68934 O69014 O69172 O69303 O69470 O69548 O70338
O70473 O74205 O75015 O76260 O76261 O76262 O76263 O76264 O76265 O76284
O76459 O77011 O77012 O77013 O77014 O77015 O77016 O77018 O77019 O77020
O77021 O77022 O77698 O77811 O81635 O83129 O83217 O83372 O83409 O83505
O83604 O83679 O83803 O83822 O83998 O84022 O84035 O84130 O84304 O84371
O84451 O84460 O84787 O84799 O84840 O84874 O85465 O86083 O86528 O87006
O88060 O88673 O88816 O88818 O93429 O94489 O94760 O97756 P00107 P00108
P00109 P00110 P00111 P00112 P00113 P00114 P00115 P00116 P00117 P00118
P00119 P00139 P00140 P00141 P00149 P00150 P00324 P00381 P00385 P00433
P00476 P00568 P00569 P00570 P00571 P00586 P00647 P00657 P00658 P00659
P00660 P00662 P00663 P00664 P00665 P00666 P00667 P00668 P00671 P00672
P00673 P00674 P00675 P00676 P00677 P00680 P00681 P00682 P00683 P00684
P00685 P00686 P00687 P00688 P00689 P00691 P00692 P00693 P00752 P00759
P00776 P00777 P00926 P00949 P00956 P00962 P01083 P01582 P02022 P02207
P02209 P02210 P02211 P02216 P02221 P02229 P02230 P02231 P02232 P02233
P02234 P02235 P02236 P02237 P02238 P02239 P02240 P02532 P02758 P02787
P02789 P02800 P02923 P02990 P02999 P03000 P04059 P04060 P04061 P04063
P04129 P04131 P04190 P04731 P04732 P04733 P04745 P04746 P04747 P04748
P04749 P04750 P04805 P04822 P04830 P05081 P05082 P05096 P05102 P05314
P05425 P05465 P05523 P05618 P05637 P05807 P05980 P06278 P06612 P06632
P07024 P07102 P07118 P07170 P07264 P07362 P07380 P07438 P07497 P07637
P07638 P07762 P07813 P07847 P07848 P07849 P07896 P07906 P07943 P08071
P08117 P08137 P08144 P08166 P08171 P08192 P08197 P08337 P08486 P08704
P08732 P08760 P08831 P09107 P09121 P09187 P09194 P09339 P09424 P09428
P09571 P09591 P09794 P09953 P09965 P09967 P09968 P10251 P10342 P10529
P10538 P10593 P10669 P10749 P10772 P10816 P10933 P11448 P11515 P11875
P11880 P11931 P11944 P11957 P12115 P12252 P12337 P12346 P12421 P12833
P13113 P13502 P13507 P13537 P13552 P13613 P13640 P13655 P13796 P13797
P13906 P13927 P14014 P14306 P14393 P14628 P14632 P14848 P14871 P14899
P14943 P14962 P15121 P15122 P15189 P15254 P15446 P15447 P15877 P15925
P16098 P16116 P16152 P16304 P16414 P16521 P16598 P16635 P16954 P16968

P17264 P17279 P17516 P17654 P17847 P17859 P17927 P18167 P18192 P18250
P18265 P18328 P18430 P18668 P18839 P18906 P19134 P19210 P19414 P19584
P19595 P19640 P19644 P19647 P19688 P19786 P19883 P19961 P20004 P20041
P20043 P20192 P20233 P20637 P20650 P20691 P20845 P21197 P21198 P21199
P21300 P21327 P21338 P21517 P21621 P21664 P21674 P21694 P21760 P21812
P21888 P22035 P22088 P22249 P22250 P22299 P22343 P22394 P22431 P22487
P22643 P22679 P22783 P22806 P22998 P23225 P23329 P23386 P23457 P23486
P23539 P23568 P23671 P23742 P23743 P24232 P24323 P24329 P24397 P24497
P24557 P24627 P24717 P24930 P25324 P25516 P25518 P25891 P25892 P25910
P25935 P26184 P26276 P26353 P26499 P26501 P26509 P26612 P26613 P26622
P26690 P26889 P26918 P26935 P27000 P27028 P27036 P27142 P27143 P27144
P27195 P27199 P27246 P27350 P27425 P27800 P27932 P27933 P27934 P27935
P27937 P27939 P27941 P27992 P27993 P28010 P28596 P29026 P29253 P29268
P29279 P29287 P29347 P29410 P29411 P29542 P29543 P29544 P29551 P29750
P29928 P29957 P29964 P30008 P30044 P30103 P30270 P30537 P30538 P30539
P30752 P30768 P30792 P30863 P30920 P30921 P31101 P31226 P31228 P31230
P31416 P31417 P31501 P31514 P31515 P31746 P31747 P31797 P31835 P31970
P32000 P32021 P32113 P33107 P33164 P33165 P33166 P33167 P33168 P33169
P33170 P33171 P33221 P33318 P33319 P33320 P33321 P33655 P33685 P33686
P33687 P33688 P33918 P34184 P34185 P34346 P34455 P34802 P35003 P35140
P35141 P35167 P35398 P35493 P35494 P35507 P35509 P35640 P35644 P35813
P35814 P35815 P35868 P36222 P36420 P36423 P36432 P36623 P36871 P36924
P36982 P36993 P37012 P37032 P37302 P37330 P37352 P37407 P37416 P37417
P37689 P37725 P37880 P37891 P37896 P37897 P37967 P38372 P38500 P38505
P38552 P38652 P38998 P39068 P39069 P39118 P39286 P39701 P39771 P39773
P39814 P39915 P39917 P40106 P40174 P40175 P40371 P40686 P40874 P41208
P41209 P41257 P41259 P41260 P41277 P41368 P41509 P41687 P41914 P41972
P42158 P42371 P42439 P42450 P42471 P42472 P42473 P42474 P42475 P42476
P42477 P42478 P42479 P42480 P42481 P42482 P42593 P42812 P43012 P43065
P43379 P43412 P43414 P43417 P43807 P43814 P43816 P43818 P43824 P43831
P43832 P43834 P43847 P43905 P43926 P44318 P44319 P44330 P44421 P44932
P44948 P45061 P45177 P45263 P45339 P45376 P45377 P45631 P45741 P45840
P45876 P46148 P46199 P46207 P46213 P46214 P46215 P46241 P46243 P46363
P46445 P46635 P46647 P46648 P46797 P46799 P46844 P46906 P47243 P47267
P47368 P47417 P47418 P47438 P47492 P47495 P47576 P47587 P47618 P47669
P47700 P47708 P47709 P47727 P47762 P47844 P47929 P47931 P47967 P47982
P48089 P48090 P48445 P48516 P48730 P48758 P48864 P48865 P49015 P49068
P49430 P49441 P49442 P49443 P49589 P49601 P49609 P49674 P49840 P49924
P49973 P49974 P49979 P49980 P49981 P50062 P50063 P50064 P50065 P50066
P50067 P50068 P50070 P50107 P50291 P50578 P51003 P51004 P51005 P51006
P51163 P51200 P51231 P51375 P51448 P51493 P51556 P51635 P51652 P51698
P51745 P52196 P52308 P52310 P52312 P52390 P52640 P52706 P52707 P52832
P52834 P52854 P52897 P52979 P52980 P52981 P53004 P53398 P53441 P53635
P53636 P53662 P54136 P54215 P54220 P54545 P54560 P54819 P55044 P55045

P55251 P55262 P55264 P55811 P55872 P55973 P55991 P56000 P56003 P56064
P56102 P56104 P56120 P56128 P56196 P56197 P56218 P56271 P56410 P56456
P56470 P56534 P56634 P56690 P56927 P56952 P57000 P57014 P57024 P57028
P57029 P57067 P57068 P57069 P57070 P57164 P57173 P57210 P57226 P57249
P57298 P57315 P57371 P57396 P57447 P57490 P57556 P57558 P57736 P57813
P57834 P57835 P57837 P57847 P57851 P57890 P57906 P57910 P57939 P57966
P57990 P58117 P58280 P58523 P58744 P58963 P58964 P58965 P59065 P59076
P59078 P59081 P59173 P59174 P59175 P59176 P59177 P59336 P59337 P59416
P59421 P59423 P59429 P59434 P59446 P59477 P59483 P59506 P59663 P59690
P59691 P59742 P59743 P59816 P59945 P59946 P59952 P61821 P61822 P61823
P61824 P62632 P63033 P63584 P63585 P63962 P63963 P63964 P63965 P63985
P63986 P63987 P63988 P63989 P63990 P63991 P63992 P64024 P64025 P64026
P64027 P64028 P64029 P64030 P64031 P64148 P64149 P64150 P64151 P64152
P64153 P64154 P64155 P64269 P64270 P64301 P64302 P64303 P64304 P65135
P65136 P65137 P65138 P65139 P65140 P65141 P65142 P65143 P65144 P65145
P65146 P65147 P65148 P65201 P65202 P65203 P65204 P65840 P65841 P66673
P66674 P67017 P67018 P67021 P67022 P67023 P67024 P67508 P67509 P67569
P67570 P67599 P67600 P67678 P67679 P67680 P67681 P67682 P67683 P67684
P67685 P67926 P67927 P67928 P67929 P67982 P67983 P69882 P70052 P70473
P70694 P70705 P70920 P71481 P72231 P72483 P72641 P72874 P73302 P73505
P73810 P74143 P74227 P74290 P74330 P74507 P74759 P74871 P74881 P74893
P75022 P75091 P75114 P75167 P75222 P75258 P75304 P75402 P75423 P75523
P77986 P78032 P78368 P78722 P79161 P79162 P79182 P79340 P79376 P79377
P79378 P79379 P79380 P79431 P79774 P79815 P79819 P80015 P80018 P80036
P80042 P80098 P80099 P80219 P80246 P80247 P80248 P80249 P80276 P80287
P80289 P80290 P80291 P80292 P80294 P80295 P80296 P80297 P80298 P80370
P80425 P80426 P80429 P80494 P80495 P80561 P80721 P80722 P81030 P81031
P81123 P81245 P81594 P81638 P81641 P81661 P81901 P81902 P81926 P82447
P82462 P82463 P82464 P82559 P82809 P83039 P83052 P83053 P83391 P83409
P83682 P83833 P87351 P91778 P94186 P94701 P95479 P95724 P95744 P95896
P96022 P96142 P96551 P96862 P97584 P97590 P99029 P99062 P99152 P99170
Q00464 Q00972 Q00985 Q01234 Q01373 Q01401 Q01698 Q01770 Q02198 Q02207
Q02418 Q03321 Q03366 Q03421 Q03464 Q03469 Q03710 Q04446 Q04505 Q04519
Q04570 Q04828 Q05192 Q05426 Q05487 Q05489 Q05506 Q05615 Q05639 Q05873
Q05932 Q06434 Q06486 Q06752 Q07006 Q07705 Q07744 Q07801 Q08047 Q08079
Q08258 Q08341 Q08346 Q08426 Q08582 Q08662 Q08751 Q08885 Q08888 Q09099
Q09139 Q09145 Q09163 Q09172 Q09173 Q09790 Q10276 Q10625 Q10730 Q10768
Q11010 Q11132 Q12798 Q14651 Q14914 Q16613 Q16762 Q16775 Q17058 Q20140
Q23834 Q23835 Q24956 Q26998 Q27177 Q27178 Q28292 Q28385 Q28386 Q28579
Q28719 Q28960 Q29058 Q29073 Q29443 Q29477 Q29495 Q29545 Q29606 Q29614
Q39017 Q39161 Q42908 Q43133 Q43270 Q44315 Q44532 Q44683 Q45234 Q45477
Q45560 Q46444 Q46550 Q46857 Q46930 Q46978 Q47066 Q47155 Q49006 Q49784
Q49988 Q50299 Q50319 Q50596 Q50642 Q51770 Q52107 Q53042 Q53871 Q54463
Q54869 Q55486 Q55522 Q55729 Q55778 Q55801 Q56070 Q57366 Q57931 Q58477

Q58663 Q58907 Q59178 Q59190 Q59495 Q59509 Q59567 Q59591 Q59594 Q59596
Q59615 Q59627 Q59628 Q59831 Q59832 Q59833 Q59938 Q59975 Q60112 Q60480
Q61183 Q61233 Q62761 Q62762 Q62763 Q63264 Q63598 Q63764 Q64104 Q64430
Q64640 Q64666 Q66DP5 Q6FU27 Q6G792 Q6G8P5 Q6G998 Q6GBM1 Q6GBT9 Q6GBV8
Q6GBW0 Q6GEK4 Q6GG25 Q6GGU5 Q6GJ61 Q6GJC0 Q6GJD9 Q6GJE1 Q6ITZ4 Q6Q3H0
Q70I53 Q71V39 Q72M46 Q75BV4 Q7M7L9 Q7M7W9 Q7M8A5 Q7M9L9 Q7MGZ2 Q7MII6
Q7MLR5 Q7MMM3 Q7MMQ4 Q7MMR5 Q7MMW8 Q7MPS3 Q7MUF7 Q7MVQ6 Q7MW54 Q7MY36
Q7N0P5 Q7N565 Q7N6Y2 Q7N743 Q7NAE5 Q7NAQ5 Q7NC67 Q7NFW7 Q7NI06 Q7NJS6
Q7NKT5 Q7NMK9 Q7NQS5 Q7NSR8 Q7NSS7 Q7NWM1 Q7NWP4 Q7NX86 Q7NYC5 Q7SFX6
Q7TU24 Q7TUT7 Q7TV76 Q7U3V8 Q7U4I1 Q7U4V2 Q7U581 Q7U8U2 Q7UA08 Q7UFG7
Q7UJ17 Q7UN00 Q7UNF9 Q7URC7 Q7UUZ6 Q7UX42 Q7UZW3 Q7V051 Q7V2K3 Q7V2X4
Q7V396 Q7V493 Q7V526 Q7V5U5 Q7V8Y5 Q7V9N2 Q7V9Y1 Q7VA78 Q7VDB2 Q7VDK6
Q7VE03 Q7VEJ4 Q7VFA6 Q7VGZ8 Q7VHN8 Q7VJ08 Q7VKS0 Q7VL53 Q7VLM3 Q7VM15
Q7VMA0 Q7VMF1 Q7VMY0 Q7VN67 Q7VNZ3 Q7VP38 Q7VR70 Q7VRB6 Q7VRU3 Q7VUG7
Q7VUU5 Q7VY63 Q7W0K0 Q7W185 Q7W2Q2 Q7W3X9 Q7W7G0 Q7W911 Q7WDQ0 Q7WFA9
Q7WKF6 Q7WKU8 Q7Z9L3 Q812P6 Q814C4 Q814Q8 Q815K7 Q816G6 Q817G5 Q818U9
Q819U6 Q81J22 Q81J59 Q81J61 Q81JT7 Q81K82 Q81L04 Q81L15 Q81M86 Q81R81
Q81VQ9 Q81VT2 Q81VV1 Q81VV3 Q81WH7 Q81X77 Q81ZS7 Q81ZU6 Q821U7 Q822B2
Q822G0 Q822I4 Q822N0 Q822Z1 Q823Y5 Q824H4 Q828D2 Q82DM5 Q82E68 Q82JR3
Q82JU0 Q82LC4 Q82S12 Q82U77 Q82XC1 Q82Y12 Q82ZE1 Q831N1 Q837C9 Q837G3
Q839E3 Q839V7 Q83A98 Q83BH2 Q83BL6 Q83C11 Q83E75 Q83EV3 Q83G05 Q83GP4
Q83GT2 Q83HH0 Q83HJ1 Q83I60 Q83IK0 Q83JB1 Q83JC4 Q83K84 Q83KQ3 Q83L38
Q83LY4 Q83PV3 Q83RT9 Q85G77 Q864V9 Q864W0 Q864W1 Q865X7 Q865X8 Q870G1
Q877P8 Q877T5 Q879M4 Q87AB3 Q87AH7 Q87AQ6 Q87B73 Q87BU2 Q87C76 Q87DL0
Q87DN2 Q87DU6 Q87EL7 Q87ES6 Q87F22 Q87F36 Q87FP9 Q87FR0 Q87KZ5 Q87L00
Q87MB4 Q87MG2 Q87QJ9 Q87QP3 Q87QX9 Q87RD6 Q87RG4 Q87RH4 Q87RL6 Q87RN6
Q87RW0 Q87T81 Q87V03 Q87VI5 Q87Y22 Q87YQ1 Q87YQ2 Q87YT0 Q87Z72 Q881X0
Q884C8 Q886R8 Q886W6 Q888C4 Q889X3 Q88AB2 Q88AH6 Q88C93 Q88CQ5 Q88CU1
Q88CX4 Q88DC4 Q88FF5 Q88FN1 Q88IU4 Q88IU5 Q88K26 Q88LF6 Q88NK4 Q88P16
Q88PX4 Q88QN7 Q88QP8 Q88QX8 Q88V07 Q88VE0 Q88VL2 Q88WF3 Q88WK9 Q88WU8
Q88WV4 Q88X53 Q88XW5 Q88YX9 Q88YY0 Q890J1 Q890Q5 Q891T1 Q894D2 Q895P5
Q896M5 Q896N5 Q898R1 Q89AD4 Q89AG3 Q89AQ1 Q89AU9 Q89AX4 Q89B09 Q89FD3
Q89KR5 Q89KR6 Q89UE3 Q89WC9 Q89WH7 Q89ZJ0 Q8A287 Q8A3X5 Q8A455 Q8A5I9
Q8A8H7 Q8AAP1 Q8BLI0 Q8BTH8 Q8CNP3 Q8CP23 Q8CPY4 Q8CQ81 Q8CRI0 Q8CS75
Q8CSI1 Q8CSV8 Q8CSX1 Q8CTN9 Q8CTU1 Q8CTU3 Q8CW45 Q8CWN5 Q8CWQ7 Q8CZE8
Q8D227 Q8D240 Q8D264 Q8D2V9 Q8D372 Q8D375 Q8D3B9 Q8D3D3 Q8D4P0 Q8D4Q0
Q8D8R1 Q8DBD5 Q8DBI7 Q8DCQ7 Q8DCV7 Q8DCW1 Q8DDY4 Q8DF81 Q8DFH5 Q8DFM1
Q8DFR1 Q8DHW6 Q8DIG8 Q8DK79 Q8DKN4 Q8DLB8 Q8DLI5 Q8DLY3 Q8DML4 Q8DN69
Q8DPS6 Q8DQ33 Q8DQS8 Q8DRD4 Q8DRW2 Q8DS33 Q8DT52 Q8DUV8 Q8DV22 Q8DVW1
Q8DVX9 Q8DWA9 Q8DWV9 Q8DXR9 Q8E081 Q8E0U0 Q8E1Z4 Q8E284 Q8E2R1 Q8E3D9
Q8E5V8 Q8E6F8 Q8E7F2 Q8E7P2 Q8E8D9 Q8E9Y7 Q8EC57 Q8EE30 Q8EEH8 Q8EER8
Q8EFF5 Q8EG22 Q8EG26 Q8EGU7 Q8EJ79 Q8EM45 Q8EPE6 Q8EPF5 Q8EQ56 Q8EQC1
Q8ER21 Q8ETB7 Q8ETW3 Q8ETY4 Q8ETZ9 Q8EU02 Q8EU94 Q8EUD3 Q8EUL6 Q8EW33
Q8EX18 Q8EYM3 Q8EZ61 Q8F525 Q8F5J3 Q8F6P5 Q8F6Q9 Q8FAL3 Q8FC87 Q8FCA6

Q8FCR7 Q8FD13 Q8FDN6 Q8FF26 Q8FFC9 Q8FGQ0 Q8FIR3 Q8FJB6 Q8FJW4 Q8FJY9
Q8FK44 Q8FK84 Q8FMI8 Q8FNB3 Q8FP17 Q8FPU9 Q8FQ12 Q8FQ57 Q8FRI2 Q8FS39
Q8FTQ2 Q8FW50 Q8FXR6 Q8FZ50 Q8G092 Q8G0E8 Q8G0S1 Q8G146 Q8G3C4 Q8G3Z8
Q8G4V2 Q8G5L0 Q8G709 Q8GF77 Q8GGL2 Q8GQC5 Q8K419 Q8K5J2 Q8K5T7 Q8K5Y2
Q8K719 Q8K872 Q8K8D0 Q8K8P9 Q8K8X1 Q8K980 Q8K9E1 Q8K9I1 Q8K9P7 Q8K9U2
Q8K9Z2 Q8KA47 Q8KAH0 Q8KAK2 Q8KAM9 Q8KB71 Q8KC52 Q8KD69 Q8KGF3 Q8KI92
Q8KPU9 Q8KT95 Q8KT97 Q8KT99 Q8KTA1 Q8KTA3 Q8KTA6 Q8L1E5 Q8L2S1 Q8NL22
Q8NMD7 Q8NNP4 Q8NNV7 Q8NQP8 Q8NQX9 Q8NR40 Q8NRZ4 Q8NWB7 Q8NWN5 Q8NX29
Q8NXL5 Q8NXT8 Q8NXY7 Q8NZ22 Q8NZD1 Q8NZI1 Q8P0H1 Q8P1W4 Q8P250 Q8P2N7
Q8P2Z4 Q8P3C4 Q8P455 Q8P5H9 Q8P5P5 Q8P7C5 Q8P7Z3 Q8P8J0 Q8P991 Q8PA95
Q8PBX8 Q8PC51 Q8PC59 Q8PC65 Q8PCB3 Q8PCQ7 Q8PCW8 Q8PDD1 Q8PE48 Q8PEQ4
Q8PFR2 Q8PGJ4 Q8PGR7 Q8PGW9 Q8PH23 Q8PIP8 Q8PJE2 Q8PK23 Q8PKZ8 Q8PLY5
Q8PNH8 Q8PNU2 Q8PNZ5 Q8PQA2 Q8PR13 Q8PT42 Q8PTZ6 Q8PV26 Q8PXI0 Q8PYH6
Q8QGR0 Q8R164 Q8R5Y3 Q8R603 Q8R685 Q8R786 Q8R7T1 Q8R7T3 Q8R7T8 Q8R7V2
Q8R7X4 Q8R979 Q8R9C2 Q8R9T7 Q8RA67 Q8RB11 Q8RB93 Q8RDZ8 Q8RE31 Q8REB0
Q8RF62 Q8RG14 Q8RIK9 Q8RLV9 Q8RQ01 Q8SPN3 Q8SPN4 Q8SPN5 Q8SPQ0 Q8SQ05
Q8SQ06 Q8SQ07 Q8SQ09 Q8SQ11 Q8SQ13 Q8SQ14 Q8SV03 Q8THH3 Q8TIW3 Q8TRS3
Q8TXN4 Q8U0A0 Q8U207 Q8U671 Q8U7H5 Q8U8L4 Q8UAA5 Q8UE16 Q8UE38 Q8UEP4
Q8UFA2 Q8UFV3 Q8UGG4 Q8UH04 Q8UHA7 Q8UIH4 Q8UJ85 Q8UJK7 Q8VCT3 Q8VP65
Q8VW76 Q8W1L6 Q8WKJ8 Q8X6X6 Q8X711 Q8X7Q1 Q8X7Z7 Q8X839 Q8X8F1 Q8X9H8
Q8XA15 Q8XA46 Q8XBN2 Q8XBT6 Q8XCH2 Q8XCK5 Q8XCT9 Q8XDE9 Q8XDG9 Q8XDP1
Q8XFP8 Q8XGZ0 Q8XHQ5 Q8XHU4 Q8XJ67 Q8XJL9 Q8XJU2 Q8XK15 Q8XK37 Q8XKU2
Q8XMJ2 Q8XMP3 Q8XPA2 Q8XPV7 Q8XT76 Q8XWE1 Q8XZ19 Q8XZ28 Q8XZ91 Q8Y065
Q8Y077 Q8Y0V3 Q8Y0Y6 Q8Y199 Q8Y2D7 Q8Y2P8 Q8Y422 Q8Y449 Q8Y493 Q8Y4I4
Q8Y5T0 Q8Y5Y0 Q8Y680 Q8Y6W7 Q8Y7F0 Q8YAB1 Q8YAB3 Q8YC76 Q8YE68 Q8YED2
Q8YEG1 Q8YG89 Q8YGR9 Q8YH17 Q8YH36 Q8YHG4 Q8YHL9 Q8YIR3 Q8YK41 Q8YMB5
Q8YNE3 Q8YP63 Q8YP80 Q8YPJ8 Q8YPL2 Q8YQU9 Q8YS86 Q8YUA3 Q8YXW5 Q8YYX9
Q8Z068 Q8Z0M3 Q8Z193 Q8Z1X0 Q8Z235 Q8Z2F0 Q8Z2H2 Q8Z2L4 Q8Z4L6 Q8Z4X1
Q8Z5V7 Q8Z6A0 Q8Z6I3 Q8Z813 Q8Z8F8 Q8Z8P6 Q8Z988 Q8ZA75 Q8ZAN8 Q8ZBZ9
Q8ZCC0 Q8ZCK0 Q8ZCQ2 Q8ZDD9 Q8ZDW6 Q8ZEV7 Q8ZEY0 Q8ZGC6 Q8ZH30 Q8ZH36
Q8ZI40 Q8ZIX0 Q8ZJB2 Q8ZJN0 Q8ZJP0 Q8ZKB0 Q8ZKF6 Q8ZL08 Q8ZL56 Q8ZLG5
Q8ZLM6 Q8ZM06 Q8ZQD4 Q8ZQX5 Q8ZR68 Q8ZRM7 Q8ZXT5 Q90844 Q90W04 Q90YJ2
Q91053 Q91379 Q91YJ5 Q91Z53 Q92021 Q92079 Q920N2 Q920P5 Q921I1 Q925Y6
Q927I6 Q927M8 Q927T2 Q928I2 Q92A40 Q92A85 Q92AI9 Q92BF1 Q92BQ5 Q92C22
Q92F36 Q92F38 Q92GL5 Q92GS6 Q92GT4 Q92GW4 Q92GY7 Q92HK6 Q92IH1 Q92IH2
Q92IV0 Q92JF6 Q92JH2 Q92M14 Q92PX0 Q92Q31 Q92QM8 Q92R20 Q92RG0 Q92ST3
Q92SV5 Q92TA4 Q92XH8 Q93083 Q932F6 Q932G0 Q937W7 Q93ED4 Q93FE6 Q93HU3
Q93K00 Q93PU8 Q96D21 Q96Y91 Q971T7 Q975P6 Q97ED6 Q97EH5 Q97EJ9 Q97GK5
Q97IC1 Q97K78 Q97KC9 Q97KM2 Q97L53 Q97MB3 Q97NG1 Q97QS8 Q97R61 Q97S25
Q97SC7 Q97SU1 Q97W02 Q97ZF5 Q97ZZ8 Q980I5 Q98157 Q981F7 Q984X8 Q985P4
Q985W1 Q98BG6 Q98C03 Q98CC1 Q98CP5 Q98DK4 Q98JM4 Q98JM5 Q98LC0 Q98LV1
Q98LZ1 Q98MD0 Q98MV8 Q98N36 Q98NW7 Q98PN8 Q98Q02 Q98QA7 Q98QB3 Q98QG1
Q98QQ1 Q98QV2 Q99798 Q99JA8 Q99QM0 Q99W05 Q99W73 Q99XL5 Q99XZ9 Q99Y66
Q99Z83 Q9A131 Q9A1H9 Q9A259 Q9A2H2 Q9A341 Q9A347 Q9A5I1 Q9A721 Q9A884

Q9A8T2 Q9A9D9 Q9AAY2 Q9AE55 Q9AGV2 Q9BGI1 Q9BN01 Q9BW91 Q9CC22 Q9CCG2
Q9CCI3 Q9CD55 Q9CDZ7 Q9CE12 Q9CE21 Q9CEB7 Q9CEI0 Q9CEJ0 Q9CEJ7 Q9CEU0
Q9CG80 Q9CIK4 Q9CIQ1 Q9CLI9 Q9CLW4 Q9CMD1 Q9CN30 Q9CN42 Q9CN94 Q9CNG4
Q9CWS0 Q9CY64 Q9D0F9 Q9D0I9 Q9D7Q1 Q9DBM2 Q9DC28 Q9DEQ3 Q9EQH2 Q9ER97
Q9EV05 Q9F1L9 Q9F2I9 Q9F724 Q9FK25 Q9GLW7 Q9GLW9 Q9GQV3 Q9HCP0 Q9HLE6
Q9HPA7 Q9HQC1 Q9HQT4 Q9HU53 Q9HUC8 Q9HUL3 Q9HXN2 Q9HXV4 Q9HZJ5 Q9I0A0
Q9I0M4 Q9I1W2 Q9I2S9 Q9I2U7 Q9I2U8 Q9I534 Q9I636 Q9JII6 Q9JJ22 Q9JMK2
Q9JN65 Q9JRG1 Q9JSM2 Q9JTD9 Q9JTM7 Q9JTT3 Q9JWC5 Q9JWJ3 Q9JWT4 Q9JX22
Q9JXE6 Q9JXK5 Q9JYE5 Q9JYM8 Q9JYS8 Q9JYU1 Q9K1A4 Q9K1H7 Q9K1R6 Q9K303
Q9K4A7 Q9K6C1 Q9K716 Q9K855 Q9K867 Q9K8G8 Q9K9A8 Q9K9Z1 Q9KA23 Q9KB03
Q9KCA6 Q9KCU7 Q9KD44 Q9KEL8 Q9KGF4 Q9KGF6 Q9KJA7 Q9KJA8 Q9KJA9 Q9KJB0
Q9KNE8 Q9KP73 Q9KPS5 Q9KPX8 Q9KQC6 Q9KQZ9 Q9KRB0 Q9KRB2 Q9KTA6 Q9KTB7
Q9KTN2 Q9KUZ6 Q9KV18 Q9KV22 Q9KV37 Q9KVC5 Q9KX08 Q9L0Q6 Q9L213 Q9L214
Q9L6L9 Q9L6Q3 Q9L7T2 Q9L9D7 Q9MS97 Q9NJN7 Q9NJN8 Q9NJP0 Q9NPG2 Q9NQ30
Q9NR19 Q9NUT2 Q9NZ08 Q9P9Q9 Q9PA74 Q9PB21 Q9PBI6 Q9PDC7 Q9PDF6 Q9PDP1
Q9PEN3 Q9PEV8 Q9PF56 Q9PFE1 Q9PFV1 Q9PGM3 Q9PGZ9 Q9PH12 Q9PHK2 Q9PHM8
Q9PI71 Q9PIS2 Q9PJT8 Q9PJU7 Q9PK28 Q9PK73 Q9PK91 Q9PL07 Q9PL20 Q9PL51
Q9PL86 Q9PLC9 Q9PLE0 Q9PLZ2 Q9PM37 Q9PM39 Q9PP78 Q9PPB8 Q9PPE4 Q9PPP0
Q9PQ76 Q9PQL3 Q9PQM4 Q9PQP0 Q9PQR4 Q9PQS5 Q9PQU6 Q9PQW1 Q9PR21 Q9PSN1
Q9Q8Z2 Q9QB93 Q9QXY8 Q9QYX2 Q9QYX3 Q9R063 Q9R0A9 Q9R0Y5 Q9R1K9 Q9R342
Q9RB01 Q9RN37 Q9RND7 Q9RPU5 Q9RQI5 Q9RRC4 Q9RSK7 Q9RSN7 Q9RTB7 Q9RTT6
Q9RVD3 Q9RWR5 Q9RX22 Q9RX30 Q9RY06 Q9S1N4 Q9S400 Q9TLW1 Q9TLX8 Q9TUM0
Q9U2M7 Q9UBQ7 Q9UIJ7 Q9UYT0 Q9UYT4 Q9UZ86 Q9UZN1 Q9V072 Q9W727 Q9WTP6
Q9WTP7 Q9WTT5 Q9WUR3 Q9WUR4 Q9WUS1 Q9WUS2 Q9WUS3 Q9WUV3 Q9WUV4 Q9WUV5
Q9WUX3 Q9WUX4 Q9WUX6 Q9WVG1 Q9WX29 Q9WYI0 Q9WZH8 Q9X0H8 Q9X172 Q9X1G3
Q9X1I8 Q9X1S6 Q9X242 Q9X2D7 Q9X2I8 Q9X3X1 Q9X3X7 Q9X4D0 Q9X4H2 Q9X519
Q9X5M0 Q9X6E7 Q9X909 Q9X981 Q9XB14 Q9XBN7 Q9XCL6 Q9XD15 Q9XDB4 Q9XS77
Q9XST5 Q9Y258 Q9Y466 Q9Y6K5 Q9Y6K8 Q9Y6M4 Q9YC75 Q9YCB6 Q9YGD3 Q9YGL3
Q9YHV4 Q9Z377 Q9Z3T0 Q9Z3U0 Q9Z409 Q9Z470 Q9Z612 Q9Z6M0 Q9Z6R9 Q9Z6W4
Q9Z6X4 Q9Z7Y3 Q9Z7Z3 Q9Z876 Q9Z8U0 Q9Z959 Q9Z972 Q9Z987 Q9Z9A7 Q9Z9L6
Q9ZBQ6 Q9ZBT9 Q9ZCK3 Q9ZCN6 Q9ZCP0 Q9ZCS6 Q9ZCT8 Q9ZCU4 Q9ZD19 Q9ZDK2
Q9ZDK3 Q9ZDT9 Q9ZE62 Q9ZE81 Q9ZER0 Q9ZEU3 Q9ZFA3 Q9ZFF7 Q9ZHB3 Q9ZHD7
Q9ZJJ1 Q9ZJT0 Q9ZK19 Q9ZK61 Q9ZKF7 Q9ZKM7 Q9ZKW6 Q9ZL58 Q9ZLH3 Q9ZLJ1
Q9ZLL8 Q9ZLZ7 Q9ZMB9 Q9ZMV2 Q9ZMV7 Q9ZN49

Swissprot Accession Number of 5061 Homodimers

O00084 O00087 O00154 O00469 O00764 O01824 O02611 O02640 O02768 O04385
O04895 O04986 O04996 O05204 O05814 O05940 O05947 O06465 O06591 O06622
O06941 O07118 O07131 O07151 O07184 O07657 O07896 O08349 O08357 O08359
O08384 O08564 O08590 O08600 O08689 O08709 O08710 O08749 O08914 O09131
O13285 O13505 O13739 O14018 O14429 O14494 O14793 O15217 O15382 O16140
O16170 O16172 O18480 O18750 O18828 O18830 O18831 O18836 O18879 O18956
O19006 O19021 O19053 O19183 O22373 O22668 O22682 O23627 O23733 O23735

O23760 O24047 O24174 O24308 O24364 O24876 O25121 O25165 O25296 O25528
O25627 O25655 O25801 O26156 O26232 O26334 O26687 O26819 O27199 O27350
O27375 O27859 O27888 O27995 O28034 O28429 O28533 O28819 O29049 O29323
O29333 O29362 O29610 O30394 O30397 O30706 O30862 O30973 O31060 O31158
O31168 O31219 O31269 O31292 O31751 O31755 O32038 O32039 O32481 O32757
O32823 O33012 O33117 O33174 O33380 O33822 O33921 O33925 O34002 O34092
O34247 O34324 O34443 O34453 O35078 O35111 O35244 O35251 O35312 O35331
O35485 O35660 O35757 O35854 O35855 O42130 O42220 O42221 O42222 O42581
O42582 O42583 O42724 O43056 O43688 O43708 O43915 O46412 O46560 O46564
O46576 O46649 O46650 O48902 O48905 O48929 O49044 O49073 O49187 O49230
O49934 O50311 O50657 O51038 O51128 O51146 O51244 O51343 O51344 O51402
O51662 O51696 O51718 O52534 O52537 O52540 O52633 O52765 O52831 O53345
O53366 O53717 O54367 O54592 O54754 O55023 O57380 O57740 O58320 O58462
O58489 O58721 O58776 O58855 O58883 O58885 O59023 O59258 O59291 O59402
O59651 O59924 O59930 O60027 O60542 O60568 O60825 O61078 O62664 O62698
O62725 O62768 O62770 O62812 O65174 O65768 O66042 O66108 O66440 O66522
O66557 O66601 O66607 O66608 O66647 O66662 O66686 O66745 O66790 O66990
O66998 O67036 O67258 O67291 O67520 O67583 O67589 O67632 O67716 O67742
O67781 O67857 O68446 O68873 O69054 O69056 O69267 O69297 O69519 O70250
O70300 O70301 O73682 O73872 O74025 O74110 O74237 O75390 O75874 O76511
O77588 O80433 O81122 O81154 O81155 O82030 O82059 O82399 O82436 O82827
O82864 O83245 O83548 O83612 O83640 O83647 O83653 O83668 O83728 O83790
O83796 O83806 O83809 O83949 O83950 O84001 O84065 O84081 O84101 O84125
O84212 O84332 O84335 O84350 O84381 O84456 O84546 O84547 O84585 O84589
O84734 O84786 O84903 O85064 O85071 O85192 O85824 O86062 O86459 O86504
O87007 O87197 O87320 O87330 O87340 O87696 O87776 O87821 O88533 O88844
O88956 O89049 O89106 O93295 O93449 O93623 O93744 O93794 O94039 O94114
O94178 O95393 O95718 O96432 O96567 O96571 O96650 O96771 O97438 P00105
P00138 P00145 P00211 P00261 P00262 P00263 P00264 P00265 P00333 P00334
P00346 P00347 P00348 P00353 P00371 P00382 P00390 P00391 P00392 P00438
P00441 P00442 P00443 P00445 P00446 P00469 P00470 P00471 P00495 P00502
P00503 P00504 P00505 P00506 P00507 P00508 P00509 P00669 P00817 P00860
P00889 P00939 P00940 P00941 P00942 P00943 P00947 P00951 P00952 P00953
P00954 P00955 P00958 P00959 P01084 P01085 P01266 P01407 P02213 P02214
P02215 P02222 P02223 P02226 P02227 P02228 P02779 P02786 P02905 P03019
P03021 P03022 P03024 P03880 P03946 P03971 P04035 P04077 P04168 P04173
P04178 P04252 P04636 P04693 P04707 P04789 P04790 P04803 P04818 P04824
P04828 P04903 P04906 P05020 P05021 P05031 P05044 P05054 P05058 P05109
P05162 P05194 P05201 P05202 P05336 P05341 P05344 P05369 P05644 P05645
P05979 P06027 P06204 P06208 P06280 P06525 P06592 P06593 P06594 P06595
P06715 P06744 P06745 P06785 P06786 P06802 P06854 P06882 P06913 P06958
P06986 P06988 P06999 P07015 P07139 P07159 P07160 P07161 P07162 P07245
P07284 P07342 P07383 P07435 P07464 P07509 P07606 P07607 P07632 P07650
P07669 P07672 P07691 P07741 P07772 P07803 P07850 P07912 P07952 P07953

P07997 P08009 P08030 P08059 P08078 P08096 P08110 P08113 P08178 P08179
P08200 P08228 P08238 P08244 P08249 P08319 P08332 P08509 P08515 P08660
P08663 P08680 P08712 P08716 P08791 P08793 P08870 P08906 P08907 P08937
P09029 P09057 P09063 P09142 P09147 P09156 P09211 P09212 P09249 P09369
P09370 P09372 P09377 P09466 P09488 P09534 P09610 P09622 P09623 P09624
P09625 P09670 P09678 P09792 P09858 P09950 P10045 P10089 P10145 P10299
P10351 P10369 P10370 P10539 P10584 P10600 P10624 P10648 P10649 P10658
P10730 P10791 P10807 P10847 P10848 P10931 P11028 P11032 P11035 P11044
P11096 P11418 P11428 P11445 P11475 P11499 P11537 P11586 P11604 P11605
P11684 P11708 P11731 P11765 P11766 P11832 P11883 P11886 P11926 P11959
P12010 P12320 P12341 P12344 P12426 P12461 P12462 P12531 P12544 P12548
P12549 P12550 P12623 P12643 P12644 P12645 P12674 P12677 P12709 P12711
P12745 P12807 P12854 P12863 P12886 P12994 P12995 P12998 P13000 P13030
P13100 P13110 P13196 P13221 P13226 P13375 P13376 P13397 P13398 P13603
P13652 P13691 P13717 P13745 P13920 P13926 P13954 P13982 P13995 P13998
P14010 P14019 P14061 P14097 P14099 P14100 P14139 P14152 P14173 P14218
P14219 P14324 P14394 P14412 P14528 P14540 P14625 P14712 P14713 P14714
P14825 P14830 P14868 P14909 P14920 P14942 P15001 P15039 P15107 P15144
P15145 P15178 P15203 P15214 P15259 P15327 P15348 P15426 P15428 P15453
P15499 P15541 P15626 P15684 P15691 P15719 P15874 P15964 P16026 P16047
P16081 P16099 P16118 P16171 P16176 P16246 P16290 P16406 P16413 P16453
P16524 P16612 P16659 P17125 P17169 P17174 P17202 P17239 P17242 P17247
P17248 P17256 P17438 P17480 P17505 P17569 P17570 P17571 P17577 P17584
P17606 P17648 P17731 P17735 P17751 P17770 P17783 P17810 P18075 P18080
P18088 P18120 P18155 P18240 P18255 P18256 P18297 P18332 P18492 P18607
P18669 P18869 P18894 P18925 P18966 P19113 P19117 P19157 P19213 P19409
P19446 P19476 P19479 P19480 P19494 P19583 P19631 P19639 P19663 P19801
P19836 P19854 P19862 P19874 P19926 P19980 P19981 P19982 P19983 P20044
P20108 P20132 P20136 P20137 P20182 P20228 P20289 P20306 P20379 P20432
P20456 P20586 P20624 P20646 P20707 P20711 P20863 P20937 P21214 P21267
P21274 P21275 P21518 P21528 P21553 P21588 P21589 P21633 P21656 P21762
P21820 P21855 P21889 P21898 P21954 P21977 P22003 P22133 P22233 P22245
P22246 P22256 P22326 P22346 P22359 P22411 P22437 P22444 P22538 P22557
P22715 P22781 P22793 P22805 P22811 P22818 P22853 P22862 P22874 P22942
P22944 P22945 P22983 P22985 P23007 P23034 P23102 P23105 P23120 P23129
P23189 P23202 P23219 P23236 P23237 P23247 P23254 P23277 P23278 P23279
P23312 P23345 P23346 P23359 P23361 P23381 P23390 P23395 P23417 P23542
P23575 P23593 P23599 P23612 P23668 P23721 P23738 P23793 P23893 P23908
P23920 P23942 P23965 P23991 P24015 P24098 P24118 P24186 P24267 P24281
P24288 P24298 P24325 P24404 P24472 P24474 P24479 P24480 P24549 P24630
P24669 P24670 P24702 P24704 P24706 P24802 P25026 P25077 P25093 P25113
P25114 P25139 P25141 P25151 P25377 P25409 P25437 P25468 P25703 P25720
P25721 P25726 P25748 P25764 P25841 P25842 P25971 P25972 P25977 P25988
P25995 P26205 P26242 P26257 P26285 P26297 P26298 P26311 P26325 P26427

P26511 P26617 P26624 P26719 P26760 P26788 P26829 P26830 P26894 P27029
P27082 P27090 P27091 P27095 P27106 P27117 P27118 P27119 P27137 P27252
P27422 P27471 P27486 P27539 P27570 P27581 P27607 P27623 P27653 P27718
P27783 P27811 P27876 P27967 P27968 P27969 P28008 P28011 P28161 P28237
P28242 P28304 P28338 P28354 P28469 P28474 P28483 P28484 P28494 P28593
P28609 P28723 P28734 P28735 P28755 P28756 P28757 P28758 P28801 P28810
P28843 P29092 P29102 P29116 P29130 P29212 P29218 P29240 P29242 P29271
P29333 P29401 P29420 P29428 P29474 P29495 P29509 P29535 P29564 P29613
P29696 P29727 P29728 P29736 P29737 P29738 P29932 P30041 P30048 P30053
P30104 P30106 P30107 P30108 P30112 P30113 P30114 P30115 P30116 P30125
P30182 P30190 P30341 P30350 P30371 P30626 P30711 P30712 P30713 P30726
P30733 P30741 P30753 P30782 P30798 P30838 P30854 P30884 P30885 P30886
P30901 P30903 P30907 P30949 P31023 P31025 P31046 P31052 P31116 P31300
P31331 P31531 P31572 P31593 P31638 P31670 P31671 P31949 P31950 P31958
P31992 P32056 P32073 P32119 P32260 P32377 P32419 P32518 P32621 P32664
P32724 P32755 P32958 P33097 P33119 P33160 P33198 P33315 P33431 P33447
P33529 P33530 P33677 P33912 P34037 P34058 P34094 P34106 P34439 P34534
P34575 P34697 P34733 P34738 P34751 P34760 P34795 P34796 P34797 P34819
P34820 P34821 P34822 P34914 P34936 P34937 P34945 P35144 P35270 P35340
P35354 P35355 P35484 P35505 P35621 P35661 P35673 P35700 P35704 P35705
P35812 P35906 P35914 P35915 P36186 P36187 P36234 P36419 P36424 P36425
P36429 P36431 P36505 P36568 P36572 P36580 P36633 P36673 P36676 P36692
P36841 P36842 P36858 P36859 P36925 P36972 P37019 P37030 P37061 P37111
P37153 P37213 P37226 P37227 P37228 P37229 P37306 P37403 P37412 P37424
P37425 P37428 P37464 P37465 P37473 P37477 P37582 P37639 P37666 P37677
P37702 P37709 P37743 P37799 P37821 P37833 P37887 P37903 P37958 P37980
P38060 P38076 P38088 P38118 P38119 P38447 P38488 P38524 P38572 P38625
P38816 P38941 P39040 P39050 P39051 P39100 P39119 P39120 P39126 P39287
P39643 P39714 P39772 P39849 P39863 P39864 P39865 P39866 P39867 P39868
P39869 P39870 P39871 P39882 P39916 P39919 P40142 P40149 P40176 P40334
P40394 P40676 P40783 P40865 P40925 P40926 P41001 P41019 P41030 P41244
P41250 P41255 P41256 P41258 P41324 P41394 P41400 P41404 P41560 P41562
P41682 P41740 P41766 P41926 P41938 P41939 P41945 P41946 P41947 P41962
P41963 P41973 P42125 P42126 P42326 P42367 P42369 P42412 P42435 P42496
P42497 P42498 P42499 P42500 P42555 P42589 P42709 P42719 P42757 P42799
P42855 P42856 P42860 P42862 P42863 P42891 P42892 P42893 P42974 P43014
P43021 P43027 P43028 P43029 P43050 P43090 P43091 P43100 P43101 P43336
P43496 P43727 P43732 P43783 P43784 P43788 P43812 P43817 P43823 P43825
P43828 P43829 P43830 P43833 P43835 P43836 P43846 P43850 P43855 P43856
P43860 P43874 P44313 P44325 P44335 P44407 P44420 P44423 P44425 P44426
P44427 P44429 P44516 P44708 P44710 P44711 P44769 P44801 P44880 P44931
P44938 P44966 P44991 P45040 P45093 P45119 P45131 P45199 P45205 P45265
P45303 P45324 P45351 P45352 P45358 P45477 P45488 P45602 P45621 P45858
P45875 P46036 P46050 P46072 P46088 P46191 P46208 P46225 P46226 P46244

P46248 P46395 P46397 P46409 P46419 P46422 P46424 P46425 P46426 P46427
P46428 P46429 P46430 P46431 P46432 P46433 P46434 P46437 P46439 P46487
P46488 P46489 P46534 P46535 P46538 P46632 P46643 P46644 P46645 P46646
P46653 P46672 P46696 P46701 P46711 P46716 P46727 P46810 P46817 P46861
P46881 P46930 P47039 P47202 P47251 P47259 P47269 P47281 P47282 P47297
P47298 P47348 P47359 P47372 P47382 P47443 P47469 P47493 P47513 P47518
P47615 P47670 P47693 P47717 P47718 P47721 P47739 P47791 P47952 P47954
P47956 P47957 P47958 P47989 P47998 P47999 P48034 P48195 P48204 P48247
P48318 P48319 P48320 P48321 P48364 P48464 P48491 P48492 P48493 P48494
P48495 P48497 P48499 P48500 P48501 P48540 P48572 P48584 P48585 P48586
P48587 P48638 P48639 P48735 P48774 P48779 P48795 P48814 P48815 P48822
P48861 P48969 P48970 P48977 P49000 P49001 P49002 P49003 P49050 P49056
P49057 P49102 P49105 P49113 P49151 P49252 P49298 P49323 P49333 P49420
P49429 P49584 P49585 P49586 P49641 P49645 P49672 P49725 P49765 P49766
P49786 P49789 P49819 P49866 P49872 P49915 P49954 P49961 P50123 P50137
P50162 P50163 P50180 P50199 P50214 P50230 P50309 P50412 P50441 P50554
P50587 P50734 P50917 P50918 P50919 P50920 P50921 P50923 P50924 P50970
P50971 P51011 P51056 P51272 P51549 P51550 P51551 P51552 P51569 P51639
P51656 P51657 P51687 P51696 P51776 P51781 P51854 P51900 P51909 P51910
P51978 P52204 P52205 P52213 P52214 P52215 P52270 P52276 P52321 P52426
P52552 P52559 P52561 P52584 P52585 P52704 P52833 P52892 P52894 P53001
P53444 P53555 P53558 P53602 P53637 P53656 P53715 P53818 P53852 P54081
P54233 P54234 P54235 P54236 P54237 P54238 P54239 P54240 P54241 P54242
P54243 P54262 P54263 P54354 P54363 P54382 P54407 P54455 P54523 P54533
P54548 P54649 P54650 P54687 P54750 P54768 P54769 P54770 P54771 P55004
P55105 P55106 P55122 P55141 P55180 P55275 P55683 P55690 P55740 P55772
P55882 P55970 P55984 P56067 P56071 P56076 P56109 P56115 P56116 P56124
P56126 P56127 P56155 P56161 P56162 P56193 P56194 P56206 P56217 P56396
P56417 P56431 P56455 P56458 P56459 P56465 P56467 P56598 P56814 P56868
P56881 P56888 P56901 P56933 P56985 P56986 P56997 P57005 P57008 P57106
P57155 P57171 P57201 P57202 P57225 P57267 P57281 P57301 P57303 P57330
P57333 P57338 P57340 P57358 P57375 P57377 P57379 P57388 P57393 P57398
P57399 P57401 P57441 P57443 P57480 P57512 P57515 P57523 P57536 P57554
P57555 P57557 P57602 P57622 P57783 P57794 P57808 P57814 P57822 P57825
P57836 P57838 P57841 P57848 P57857 P57858 P57895 P57902 P57928 P57936
P57956 P57965 P57988 P58208 P58220 P58230 P58244 P58336 P58476 P58477
P58478 P58479 P58480 P58481 P58482 P58563 P58567 P58572 P58638 P58639
P58640 P58641 P58643 P58644 P58661 P58687 P58688 P58692 P58693 P58694
P58695 P58696 P58697 P58856 P58858 P58859 P58860 P58861 P58891 P58892
P58966 P59027 P59028 P59029 P59077 P59079 P59080 P59085 P59291 P59296
P59298 P59299 P59300 P59301 P59302 P59303 P59329 P59330 P59394 P59398
P59401 P59422 P59427 P59462 P59466 P59482 P59515 P59517 P59553 P59554
P59575 P59600 P59601 P59626 P59639 P59852 P59860 P59959 P60004 P60052
P60487 P60499 P60500 P60501 P60502 P61445 P61494 P61495 P61498 P61499

P61811 P61812 P61851 P61852 P61853 P61854 P61889 P61890 P61891 P61892
P61893 P61970 P61971 P61972 P62172 P62173 P62174 P62508 P62509 P62615
P62616 P62707 P62708 P62709 P62710 P62818 P62819 P62958 P62959 P63083
P63084 P63187 P63188 P63189 P63190 P63191 P63192 P63193 P63202 P63203
P63222 P63223 P63224 P63225 P63226 P63227 P63359 P63360 P63499 P63501
P63506 P63507 P63508 P63521 P63522 P63542 P63543 P63544 P63545 P63546
P63547 P63548 P63557 P63558 P63586 P63587 P63588 P63589 P63591 P63592
P63870 P63871 P63872 P64127 P64128 P64129 P64297 P64298 P64299 P64300
P64411 P64412 P65099 P65100 P65101 P65552 P65553 P65595 P65596 P65897
P65898 P65899 P65905 P65906 P65907 P65908 P65909 P65910 P65911 P65914
P65915 P65916 P65917 P65918 P65919 P65920 P66004 P66005 P66010 P66011
P66012 P66013 P66681 P66682 P66683 P66826 P66827 P66940 P66941 P66942
P66943 P67012 P67013 P67014 P67015 P67016 P67032 P67033 P67034 P67035
P67042 P67043 P67044 P67045 P67046 P67047 P67048 P67049 P67050 P67051
P67052 P67091 P67092 P67260 P67261 P67262 P67263 P67264 P67265 P67266
P67267 P67268 P67269 P67483 P67484 P67485 P67486 P67561 P67562 P67563
P67564 P67565 P67566 P67571 P67572 P67573 P67574 P67575 P67576 P67577
P67578 P67579 P67580 P67581 P67582 P67583 P67584 P67585 P67586 P67587
P67588 P67589 P67590 P67591 P67592 P67593 P67594 P67595 P67596 P67597
P67598 P67607 P67608 P67609 P67610 P67611 P67612 P67724 P67725 P67804
P67805 P67813 P67814 P67964 P67965 P68168 P68169 P68397 P68398 P68580
P68728 P68729 P68769 P68778 P68779 P68780 P68781 P68822 P68823 P68824
P69887 P69888 P70180 P70265 P70266 P70313 P70349 P70619 P70682 P70727
P70881 P71084 P71348 P71499 P71736 P71809 P72158 P72170 P72173 P72314
P72324 P72484 P72740 P73057 P73067 P73141 P73201 P73443 P73655 P73761
P73807 P73851 P73935 P73942 P73960 P74438 P74724 P74861 P74866 P75000
P75051 P75052 P75068 P75069 P75089 P75096 P75107 P75122 P75225 P75382
P75388 P75393 P75425 P75500 P75510 P75517 P75521 P75531 P76316 P77434
P77488 P77565 P77727 P77791 P77806 P77816 P77836 P77884 P77887 P77888
P77889 P77947 P78010 P78017 P78029 P78218 P79171 P79208 P79209 P79255
P79295 P79896 P80017 P80031 P80041 P80046 P80147 P80148 P80174 P80176
P80239 P80251 P80252 P80253 P80258 P80299 P80306 P80338 P80360 P80366
P80404 P80456 P80457 P80467 P80468 P80511 P80512 P80536 P80541 P80566
P80602 P80608 P80668 P80880 P80882 P80894 P80912 P80969 P81036 P81065
P81082 P81156 P81204 P81425 P81431 P81432 P81433 P81434 P81535 P81608
P81706 P81942 P82177 P82197 P82204 P82205 P82475 P82603 P82903 P83325
P83341 P83342 P83372 P83373 P83519 P83684 P83906 P83942 P84296 P84297
P84298 P84299 P84328 P87078 P87186 P87256 P87257 P90463 P90520 P90597
P91711 P91883 P91895 P91919 P91938 P92119 P92941 P92942 P93258 P93526
P93527 P93528 P93673 P93819 P93832 P94188 P94284 P94631 P94702 P94929
P95313 P95468 P95474 P95524 P95576 P95596 P95689 P95957 P96050 P96081
P96190 P96197 P96198 P96744 P96763 P96985 P96995 P97046 P97049 P97084
P97270 P97449 P97463 P97519 P97612 P97687 P97697 P97739 P97772 P97946
P97953 P99086 P99096 P99101 P99118 P99126 P99129 P99133 P99144 P99145

P99162 P99167 Q00257 Q00285 Q00326 Q00379 Q00412 Q00472 Q00519 Q00669
Q00670 Q00671 Q00672 Q00731 Q00834 Q01061 Q01062 Q01064 Q01065 Q01066
Q01137 Q01170 Q01179 Q01237 Q01549 Q01579 Q01802 Q01879 Q01912 Q01987
Q02075 Q02079 Q02081 Q02110 Q02135 Q02143 Q02610 Q02635 Q02636 Q02809
Q02880 Q03013 Q03134 Q03217 Q03377 Q03384 Q03395 Q03426 Q03505 Q03577
Q03647 Q04120 Q04448 Q04451 Q04467 Q04473 Q04524 Q04633 Q04760 Q04797
Q04820 Q04829 Q04933 Q04944 Q05026 Q05114 Q05145 Q05329 Q05531 Q05562
Q05593 Q05683 Q05741 Q05769 Q05813 Q05927 Q06085 Q06086 Q06087 Q06088
Q06191 Q06278 Q06318 Q06429 Q06539 Q06741 Q06774 Q06826 Q06830 Q07075
Q07103 Q07108 Q07116 Q07123 Q07177 Q07182 Q07215 Q07258 Q07262 Q07264
Q07412 Q07449 Q07511 Q07588 Q07703 Q07731 Q07796 Q07891 Q07905 Q08062
Q08129 Q08392 Q08393 Q08830 Q08862 Q08863 Q09009 Q09010 Q09129 Q09187
Q09580 Q09927 Q10155 Q10334 Q10480 Q10657 Q10836 Q11129 Q11136 Q12109
Q12390 Q12545 Q12548 Q12556 Q12574 Q12630 Q13043 Q13188 Q13219 Q13255
Q13423 Q14123 Q14249 Q15181 Q15506 Q16706 Q16772 Q16773 Q16836 Q16853
Q16873 Q16877 Q16881 Q17172 Q17335 Q17745 Q19427 Q20679 Q21824 Q22067
Q22099 Q23670 Q23762 Q23763 Q24142 Q24451 Q24641 Q24735 Q25338 Q25861
Q26695 Q27404 Q27677 Q27772 Q27775 Q28007 Q28035 Q28514 Q28901 Q28943
Q28969 Q29092 Q29371 Q29373 Q29448 Q29512 Q29535 Q29538 Q29539 Q29607
Q38924 Q39242 Q39243 Q39557 Q39566 Q40024 Q40082 Q40147 Q40762 Q41046
Q42522 Q42563 Q42611 Q42612 Q42676 Q42686 Q42831 Q42881 Q42972 Q43077
Q43175 Q43199 Q43317 Q43743 Q43744 Q43779 Q43884 Q43990 Q44151 Q44297
Q44482 Q44678 Q44843 Q44951 Q45291 Q45918 Q46127 Q46158 Q46159 Q46171
Q46175 Q46509 Q46717 Q46925 Q47012 Q47258 Q47908 Q47948 Q48935 Q48979
Q49158 Q49900 Q49901 Q50000 Q50068 Q50559 Q50637 Q50649 Q50729 Q51344
Q51375 Q51422 Q51506 Q51687 Q51700 Q51772 Q51834 Q51853 Q51945 Q52069
Q52109 Q52309 Q52428 Q53479 Q53554 Q53612 Q53638 Q53647 Q53872 Q54258
Q54465 Q54975 Q55128 Q55132 Q55168 Q55317 Q55482 Q55512 Q55574 Q55624
Q55665 Q55806 Q56063 Q56093 Q56112 Q56114 Q56144 Q56213 Q56232 Q56236
Q56268 Q56415 Q56693 Q56732 Q56734 Q56738 Q56815 Q57004 Q57301 Q57452
Q57573 Q57615 Q57700 Q58097 Q58138 Q58270 Q58487 Q58509 Q58659 Q58767
Q58787 Q58885 Q58897 Q59049 Q59081 Q59097 Q59100 Q59101 Q59179 Q59182
Q59196 Q59212 Q59228 Q59240 Q59291 Q59384 Q59397 Q59408 Q59448 Q59485
Q59516 Q59557 Q59623 Q59637 Q59642 Q59654 Q59678 Q59689 Q59754 Q59787
Q59822 Q59838 Q59918 Q59940 Q59966 Q59978 Q59984 Q59985 Q59994 Q60006
Q60013 Q60034 Q60049 Q60080 Q60099 Q60151 Q60317 Q60394 Q60550 Q60651
Q60652 Q60653 Q60654 Q60682 Q60860 Q61133 Q61171 Q61425 Q61481 Q61503
Q61539 Q61941 Q62252 Q62351 Q62401 Q62AG8 Q63147 Q63321 Q63421 Q63716
Q63921 Q63CC5 Q64105 Q64329 Q64338 Q64395 Q64399 Q64413 Q64414 Q64415
Q64427 Q64437 Q64471 Q64511 Q64XI2 Q660C0 Q66C49 Q6A6Z5 Q6A910 Q6BIS1
Q6C7L4 Q6D411 Q6F6N4 Q6FR39 Q6FRI3 Q6FWL5 Q6G1E4 Q6G563 Q6G8P3 Q6G8T4
Q6G8T8 Q6G8T9 Q6G8Y6 Q6G902 Q6G960 Q6G9D4 Q6GA08 Q6GA12 Q6GAS8 Q6GAT0
Q6GB27 Q6GB56 Q6GBX1 Q6GC09 Q6GD81 Q6GEA2 Q6GG23 Q6GG69 Q6GG72 Q6GG73
Q6GGB9 Q6GGD5 Q6GGJ4 Q6GGY0 Q6GHN0 Q6GHN4 Q6GI87 Q6GI89 Q6GII7 Q6GIL6

Q6GJF4 Q6GJJ4 Q6GKT6 Q6HA24 Q6HDK6 Q6HE20 Q6HET2 Q6HJT7 Q6IT00 Q6J936
Q6KZJ4 Q6L1P9 Q6LCE4 Q6LUA8 Q6LVN7 Q6LX60 Q6LYT2 Q6M259 Q6MB27 Q6ME16
Q6MGQ3 Q6MT05 Q6NCY6 Q6NEZ0 Q6PY58 Q6T3B0 Q6TWC4 Q6YPM0 Q70JN8 Q70Q35
Q70WY9 Q711T9 Q71Y44 Q71YI5 Q71ZJ6 Q72DW7 Q72IK6 Q730M0 Q731F6 Q732I7
Q73GX9 Q73LB4 Q73Q17 Q73T78 Q73XT9 Q74H60 Q74IT5 Q74J27 Q74JN5 Q74MH2
Q750Y8 Q751L8 Q75CM8 Q75FP0 Q79V15 Q7A9L5 Q7ABI1 Q7BZ90 Q7C0D0 Q7CH40
Q7CPZ4 Q7CQ08 Q7DDM9 Q7LLZ8 Q7M7V7 Q7M7Y6 Q7M7Z0 Q7M8A6 Q7M8C4 Q7M8K2
Q7M8S6 Q7M8W8 Q7M9J5 Q7MA34 Q7MA77 Q7MAD7 Q7MAE0 Q7MAE8 Q7MAR1 Q7MB41
Q7MDF0 Q7MGT8 Q7MH15 Q7MH47 Q7MH69 Q7MIG4 Q7MIV1 Q7MIV3 Q7MJ74 Q7MK65
Q7MLS5 Q7MLV2 Q7MLX2 Q7MMR7 Q7MN49 Q7MN92 Q7MNE1 Q7MNF0 Q7MNN6 Q7MNP6
Q7MP97 Q7MPT2 Q7MSZ3 Q7MT94 Q7MTB3 Q7MU00 Q7MUV7 Q7MUX4 Q7MWI7 Q7MWL9
Q7MXJ4 Q7MXM0 Q7MY25 Q7MYB3 Q7MYD5 Q7MYW9 Q7N015 Q7N0J7 Q7N0N9 Q7N0P1
Q7N0P4 Q7N1C8 Q7N1U7 Q7N4C1 Q7N589 Q7N6E7 Q7N6S0 Q7N705 Q7N7F7 Q7N8P2
Q7NA61 Q7NAD6 Q7NAQ4 Q7NAT8 Q7NBS4 Q7NBS9 Q7NC34 Q7NCG8 Q7NDP1 Q7NDW3
Q7NFM6 Q7NG18 Q7NGZ0 Q7NHC2 Q7NHH9 Q7NJA4 Q7NK22 Q7NL03 Q7NLT1 Q7NMP9
Q7NP63 Q7NPE7 Q7NQ92 Q7NRK9 Q7NRP0 Q7NS89 Q7NSG1 Q7NTL2 Q7NUK5 Q7NVZ0
Q7NXI4 Q7NXL5 Q7NY99 Q7NYF6 Q7NZ60 Q7NZ62 Q7NZI3 Q7P0F4 Q7PBU9 Q7RTV2
Q7S2Z9 Q7TTU9 Q7TTW1 Q7TV34 Q7TYA6 Q7TYN1 Q7U272 Q7U3A4 Q7U518 Q7U6P6
Q7U7P7 Q7U804 Q7U8P3 Q7U8S4 Q7U9Q6 Q7U9X5 Q7UA53 Q7UA73 Q7UA77 Q7UAE6
Q7UF53 Q7UFY6 Q7UM95 Q7UNC3 Q7UP89 Q7UQA4 Q7UR74 Q7UWB7 Q7UXX6 Q7UYX5
Q7UZ20 Q7UZH8 Q7UZP0 Q7V0B9 Q7V0D8 Q7V0R7 Q7V0X5 Q7V102 Q7V1G6 Q7V1N4
Q7V263 Q7V286 Q7V3N7 Q7V3Q4 Q7V3X1 Q7V4P3 Q7V4S7 Q7V5W1 Q7V5Y2 Q7V608
Q7V6T7 Q7V7D2 Q7V7D8 Q7V7Q3 Q7V9A9 Q7V9C5 Q7V9C9 Q7V9I1 Q7V9Q0 Q7VAL5
Q7VAN0 Q7VAP8 Q7VAZ8 Q7VBI9 Q7VBM9 Q7VC14 Q7VC41 Q7VCG1 Q7VEH5 Q7VEJ3
Q7VEJ7 Q7VF68 Q7VF76 Q7VFD7 Q7VFL0 Q7VG34 Q7VGF2 Q7VGK6 Q7VH74 Q7VIE2
Q7VIJ7 Q7VIP6 Q7VJB1 Q7VKQ4 Q7VKV3 Q7VLE9 Q7VLK1 Q7VLR5 Q7VLU5 Q7VM20
Q7VM65 Q7VMB7 Q7VME1 Q7VMH2 Q7VN27 Q7VNF0 Q7VNP1 Q7VNP7 Q7VNZ1 Q7VP41
Q7VPB2 Q7VQW9 Q7VQX0 Q7VQX8 Q7VR39 Q7VR54 Q7VR80 Q7VRB8 Q7VRE1 Q7VRF5
Q7VRH9 Q7VRL0 Q7VRN5 Q7VRQ6 Q7VRR8 Q7VRS2 Q7VSN4 Q7VSZ0 Q7VV87 Q7VVM4
Q7VVY0 Q7VW08 Q7VW97 Q7VWL1 Q7VWL5 Q7VY13 Q7VYC6 Q7VZ37 Q7VZT5 Q7W001
Q7W089 Q7W0M8 Q7W275 Q7W2Y3 Q7W3H5 Q7W3L2 Q7W517 Q7W545 Q7W575 Q7W5Q8
Q7W5T6 Q7W6P7 Q7W6Q1 Q7W7Q0 Q7W8T6 Q7WA56 Q7WAV6 Q7WAY8 Q7WC32 Q7WCM7
Q7WCQ5 Q7WD94 Q7WDY3 Q7WEU9 Q7WEY7 Q7WGI2 Q7WGI7 Q7WHN1 Q7WHN5 Q7WJ90
Q7WK13 Q7WK46 Q7WL37 Q7WMF2 Q7WQ31 Q7WR42 Q7WS85 Q7WTB1 Q7X222 Q7X3X5
Q7X5H2 Q7YRB5 Q7YRZ7 Q7Z4P5 Q7Z7S3 Q80Y81 Q814C9 Q817I8 Q817X3 Q817X7
Q817X8 Q818R9 Q818V3 Q819S6 Q819S7 Q819Y1 Q81B71 Q81C43 Q81E05 Q81EF2
Q81FQ1 Q81G11 Q81GK5 Q81J70 Q81JA8 Q81JC4 Q81L32 Q81LI1 Q81LI6 Q81LI7
Q81LS1 Q81M54 Q81M88 Q81N41 Q81P62 Q81R23 Q81RK2 Q81SV5 Q81SY4 Q81T67
Q81TR3 Q81TS6 Q81VW3 Q81W03 Q81W17 Q81W25 Q81W73 Q81WF5 Q81WF6 Q81WL2
Q81X76 Q820K1 Q820S5 Q821H9 Q821I3 Q821P2 Q821U6 Q822E9 Q822V6 Q823S6
Q823T8 Q823U7 Q823V1 Q824H0 Q824L3 Q824R3 Q827T0 Q827T5 Q829W1 Q82AA5
Q82BS5 Q82BX8 Q82E91 Q82EQ8 Q82EU1 Q82F68 Q82FK8 Q82HM9 Q82HS2 Q82HU1
Q82IC1 Q82KS8 Q82KW8 Q82ML4 Q82SH1 Q82SQ5 Q82TD8 Q82TU1 Q82TV8 Q82TZ9
Q82VD3 Q82WB9 Q82WI6 Q82WM3 Q82X61 Q82XE0 Q82XS2 Q82XU9 Q82YY9 Q830K2

Q830U2 Q830U4 Q831K9 Q831X4 Q833I1 Q833I2 Q833J0 Q834E3 Q834G2 Q834G6
Q837B3 Q839A8 Q839J8 Q839Q8 Q83BE5 Q83BQ3 Q83BV5 Q83BZ6 Q83C80 Q83C87
Q83D84 Q83DP1 Q83E06 Q83E97 Q83EL0 Q83FJ7 Q83FN1 Q83G46 Q83GG2 Q83GJ0
Q83GZ6 Q83H72 Q83H88 Q83HA1 Q83HC3 Q83HN8 Q83HP9 Q83HR7 Q83I20 Q83ID3
Q83JA5 Q83JU6 Q83K44 Q83KJ6 Q83LD8 Q83LP0 Q83M42 Q83MJ9 Q83PR0 Q83Q04
Q83QL4 Q83RM1 Q83SE6 Q83SG2 Q83SP1 Q845V2 Q845W3 Q84BU5 Q84I51 Q84I52
Q84I53 Q877B5 Q87A77 Q87A82 Q87AB2 Q87B10 Q87B68 Q87BK6 Q87BQ1 Q87BS7
Q87BT4 Q87BW3 Q87BX8 Q87C03 Q87C26 Q87C30 Q87CK7 Q87CW0 Q87E35 Q87EB3
Q87EF0 Q87EH7 Q87EN0 Q87EQ7 Q87F16 Q87FA3 Q87GZ9 Q87J46 Q87KQ7 Q87L13
Q87LY3 Q87ME1 Q87MG7 Q87MQ1 Q87MQ3 Q87N07 Q87N49 Q87NH5 Q87PB7 Q87Q70
Q87QL0 Q87QP1 Q87QV2 Q87R16 Q87RH5 Q87RU0 Q87RX5 Q87S07 Q87S15 Q87SA0
Q87SB1 Q87SS8 Q87SU7 Q87T31 Q87T92 Q87VF3 Q87WN9 Q87WQ1 Q87WV6 Q87WW4
Q87XM1 Q87XN0 Q87Y31 Q87YZ4 Q87ZS9 Q883H9 Q883Y9 Q884C0 Q884U6 Q886N9
Q886S6 Q886X5 Q886Y9 Q889Q1 Q88BD7 Q88C92 Q88D92 Q88DP0 Q88DU1 Q88DV4
Q88F31 Q88F33 Q88FB9 Q88FQ4 Q88FT2 Q88K27 Q88LE5 Q88LW2 Q88MH6 Q88MS3
Q88NA1 Q88NJ4 Q88NV7 Q88NW7 Q88P21 Q88P86 Q88PJ6 Q88QG7 Q88SD8 Q88UE6
Q88VG6 Q88VH0 Q88VJ8 Q88VL9 Q88VQ7 Q88VQ8 Q88W05 Q88WA8 Q88WU9 Q88XZ7
Q88Y40 Q88Y74 Q88YH4 Q88YP9 Q88Z28 Q88Z60 Q88Z97 Q891C7 Q891G7 Q891J4
Q892A7 Q892B2 Q892B5 Q892Q9 Q892X7 Q894H0 Q894P6 Q895K8 Q898R2 Q89940
Q899D9 Q899G7 Q899M9 Q899T6 Q899U2 Q89A25 Q89A92 Q89A93 Q89A94 Q89AB8
Q89AC5 Q89AJ2 Q89AJ4 Q89AJ7 Q89AK4 Q89AK6 Q89AL6 Q89AN1 Q89AN7 Q89AP0
Q89AQ8 Q89AR0 Q89AW3 Q89AX7 Q89BL4 Q89BN3 Q89CK8 Q89DI2 Q89GX0 Q89KP7
Q89KU3 Q89L00 Q89MP9 Q89NF8 Q89SB5 Q89UL9 Q89W41 Q89W91 Q89X19 Q89ZN1
Q89ZV6 Q8A0C2 Q8A0U2 Q8A0Z8 Q8A1D5 Q8A1E0 Q8A2N8 Q8A525 Q8A5W4 Q8A6M0
Q8A6N7 Q8A8C4 Q8A9E3 Q8ABA8 Q8AY55 Q8AY56 Q8BTY1 Q8C6N3 Q8CGS5 Q8CJP7
Q8CJX0 Q8CMQ1 Q8CMQ2 Q8CMT6 Q8CNL2 Q8CNX4 Q8CPG7 Q8CPJ3 Q8CPJ6 Q8CPX1
Q8CPY8 Q8CQT5 Q8CQU3 Q8CQV5 Q8CRW7 Q8CS74 Q8CS95 Q8CS98 Q8CS99 Q8CSD5
Q8CSG7 Q8CSI9 Q8CSW7 Q8CT28 Q8CT69 Q8CTD5 Q8CTG8 Q8CU95 Q8CWN4 Q8CWQ4
Q8CWS5 Q8CWT4 Q8CWW2 Q8CX68 Q8CXD2 Q8CXK5 Q8D1V0 Q8D1X5 Q8D1Y2 Q8D228
Q8D229 Q8D265 Q8D2B3 Q8D2G7 Q8D2J1 Q8D2L8 Q8D2N4 Q8D2T0 Q8D2U1 Q8D2U8
Q8D2Y4 Q8D357 Q8D392 Q8D3C0 Q8D3T9 Q8D6J0 Q8D8J6 Q8D8M6 Q8D8Q1 Q8D9G5
Q8DA13 Q8DAF0 Q8DAN7 Q8DAV2 Q8DB23 Q8DB25 Q8DBC5 Q8DBF7 Q8DBX8 Q8DCN1
Q8DCQ3 Q8DCT6 Q8DD17 Q8DDX5 Q8DEC2 Q8DEE0 Q8DEQ9 Q8DER9 Q8DEZ9 Q8DF07
Q8DF59 Q8DFA3 Q8DFM0 Q8DG51 Q8DGA5 Q8DGH9 Q8DHG3 Q8DHP7 Q8DHW5 Q8DI29
Q8DIN0 Q8DJB3 Q8DJS8 Q8DKA0 Q8DKX6 Q8DL74 Q8DLB7 Q8DLK8 Q8DM42 Q8DMA9
Q8DN46 Q8DPE0 Q8DPJ4 Q8DQ87 Q8DQL5 Q8DQL6 Q8DQN1 Q8DR22 Q8DRB3 Q8DRR1
Q8DRV9 Q8DSB7 Q8DSG3 Q8DSJ7 Q8DT90 Q8DT95 Q8DTG3 Q8DTM2 Q8DTQ4 Q8DTV1
Q8DTV2 Q8DU81 Q8DUW8 Q8DVS2 Q8DVS3 Q8DW33 Q8DWP7 Q8DWV5 Q8DXD5 Q8DXW0
Q8DZ99 Q8DZA4 Q8DZQ2 Q8DZX7 Q8E0H0 Q8E0I1 Q8E259 Q8E299 Q8E2J5 Q8E2Q7
Q8E359 Q8E3H8 Q8E4W1 Q8E4W5 Q8E5F0 Q8E5M8 Q8E644 Q8E656 Q8E7L9 Q8E7Q8
Q8E9L5 Q8E9N3 Q8EC33 Q8EC52 Q8EDF0 Q8EDZ8 Q8EEE9 Q8EEI4 Q8EEQ9 Q8EEZ1
Q8EFF7 Q8EFF9 Q8EFG1 Q8EGH1 Q8EGR9 Q8EGS0 Q8EH94 Q8EHC8 Q8EHL9 Q8EI58
Q8EJR9 Q8EK12 Q8EN68 Q8ENP4 Q8EPR5 Q8EPR9 Q8EPS0 Q8EQ58 Q8EQ97 Q8EQB9
Q8EQE2 Q8EQG0 Q8EQU9 Q8ER35 Q8ER36 Q8ERU2 Q8ESS3 Q8EU10 Q8EU59 Q8EU77

Q8EUA8 Q8EUM5 Q8EUS8 Q8EUW1 Q8EUY3 Q8EUY4 Q8EV81 Q8EVV1 Q8EW01 Q8EW03
Q8EW34 Q8EWB7 Q8EWB8 Q8EWS9 Q8EWX7 Q8EXN2 Q8EY78 Q8EY85 Q8F144 Q8F153
Q8F4A2 Q8F4F4 Q8F4P5 Q8F522 Q8F5I5 Q8F5K1 Q8F6W9 Q8F9Y3 Q8FAT5 Q8FB75
Q8FB97 Q8FBT4 Q8FBX7 Q8FCI6 Q8FCI7 Q8FCR6 Q8FDZ8 Q8FE75 Q8FEY9 Q8FF72
Q8FFE8 Q8FFK8 Q8FFX8 Q8FG51 Q8FG52 Q8FGQ9 Q8FH42 Q8FH84 Q8FHP3 Q8FHU2
Q8FI04 Q8FIR2 Q8FJ91 Q8FJB1 Q8FJB7 Q8FK51 Q8FK74 Q8FKB9 Q8FL15 Q8FL76
Q8FLA4 Q8FLY5 Q8FN62 Q8FNG2 Q8FNZ1 Q8FPI2 Q8FPL0 Q8FPL5 Q8FPV5 Q8FQR4
Q8FR47 Q8FRR3 Q8FRZ3 Q8FT19 Q8FT67 Q8FTC6 Q8FU11 Q8FVF3 Q8FVH2 Q8FX93
Q8FXW9 Q8FY98 Q8G0D9 Q8G0F7 Q8G0L8 Q8G139 Q8G292 Q8G2Y6 Q8G3W7 Q8G4S8
Q8G5P4 Q8G661 Q8G6B5 Q8G6D5 Q8G6W2 Q8G7Y3 Q8G863 Q8G864 Q8GAA0 Q8GDY3
Q8GH80 Q8GKZ1 Q8GLK9 Q8GMC0 Q8GMR7 Q8GMT0 Q8GX93 Q8GYY0 Q8HXP8 Q8HXP9
Q8HXQ0 Q8HXQ1 Q8HXQ2 Q8HXQ3 Q8HXQ4 Q8HY87 Q8J0N2 Q8J0N3 Q8J0U0 Q8JFZ2
Q8JZQ5 Q8K093 Q8K183 Q8K593 Q8K5I9 Q8K5J0 Q8K5S5 Q8K5X6 Q8K5X7 Q8K635
Q8K7E6 Q8K7V3 Q8K880 Q8K8C0 Q8K8M5 Q8K941 Q8K979 Q8K981 Q8K982 Q8K9A1
Q8K9B5 Q8K9C3 Q8K9C5 Q8K9F0 Q8K9I5 Q8K9I7 Q8K9L2 Q8K9N3 Q8K9P0 Q8K9P3
Q8K9Q1 Q8K9R7 Q8K9R9 Q8K9S2 Q8K9S6 Q8K9T7 Q8K9T9 Q8K9V9 Q8K9X1 Q8KA14
Q8KA48 Q8KAN0 Q8KCD7 Q8KCH7 Q8KCM7 Q8KCT7 Q8KCW2 Q8KD01 Q8KDK5 Q8KE61
Q8KE85 Q8KES6 Q8KFI9 Q8KFM9 Q8KFQ5 Q8KFT6 Q8KFZ5 Q8KLQ0 Q8KM02 Q8KML7
Q8KZ92 Q8L1Z5 Q8L2F3 Q8L376 Q8L399 Q8MI05 Q8MKG2 Q8MKW7 Q8NJR0 Q8NJR2
Q8NJR5 Q8NJR6 Q8NKB8 Q8NLP6 Q8NM11 Q8NN33 Q8NNC1 Q8NPB2 Q8NQ07 Q8NQ19
Q8NQ39 Q8NQC0 Q8NS38 Q8NSJ4 Q8NSR1 Q8NT73 Q8NTQ9 Q8NVJ0 Q8NW61 Q8NW68
Q8NW75 Q8NWA9 Q8NWE6 Q8NWP3 Q8NWZ5 Q8NX25 Q8NX89 Q8NXI0 Q8NXN3 Q8NXZ0
Q8NY00 Q8NYY2 Q8NZ19 Q8NZ20 Q8NZB2 Q8NZH6 Q8NZN7 Q8P1A6 Q8P1C0 Q8P1D1
Q8P1W3 Q8P1X6 Q8P234 Q8P298 Q8P3D7 Q8P3Z4 Q8P469 Q8P5L1 Q8P5R4 Q8P6E0
Q8P7T0 Q8P7Z2 Q8P815 Q8P855 Q8P8F9 Q8P8Q6 Q8P8W4 Q8P9P5 Q8P9R0 Q8PA92
Q8PAC4 Q8PAL0 Q8PAV7 Q8PAY7 Q8PBW5 Q8PC25 Q8PCE7 Q8PER4 Q8PFH5 Q8PFS5
Q8PH05 Q8PH40 Q8PHU5 Q8PJ38 Q8PJE1 Q8PJG7 Q8PJK3 Q8PJY6 Q8PK88 Q8PKS5
Q8PLC6 Q8PLH2 Q8PLJ2 Q8PLY2 Q8PM16 Q8PMB1 Q8PMK9 Q8PMP0 Q8PNG1 Q8PNP8
Q8PP46 Q8PUN1 Q8PV88 Q8PVF4 Q8PVP6 Q8PYJ4 Q8PZ76 Q8Q032 Q8Q0J4 Q8Q0L5
Q8QZR1 Q8QZR5 Q8R086 Q8R4K8 Q8R5Q4 Q8R639 Q8R7N1 Q8R966 Q8R9A4 Q8R9R6
Q8R9X8 Q8RA26 Q8RAC5 Q8RAI7 Q8RAI8 Q8RAL9 Q8RB69 Q8RC63 Q8RDD1 Q8RDI5
Q8RDJ5 Q8RDK0 Q8RDM9 Q8RDR4 Q8RDX7 Q8RE08 Q8RE57 Q8RFS6 Q8RFY7 Q8RG52
Q8RG83 Q8RG88 Q8RGA3 Q8RGH4 Q8RGJ4 Q8RGJ5 Q8RGP6 Q8RH07 Q8RH11 Q8RH70
Q8RKI6 Q8T6C4 Q8TGX1 Q8TIU5 Q8TJJ1 Q8TJQ7 Q8TKW2 Q8TLK5 Q8TQR3 Q8TR73
Q8TS37 Q8TSA6 Q8TSN5 Q8TUX2 Q8TWK0 Q8TX28 Q8TXA7 Q8TXQ0 Q8TXX3 Q8TYB1
Q8U0B3 Q8U0F3 Q8U0I8 Q8U182 Q8U1U1 Q8U221 Q8U3Y2 Q8U4A0 Q8U7H6 Q8U9W3
Q8UD91 Q8UDS3 Q8UEL1 Q8UEQ2 Q8UEY3 Q8UFL9 Q8UG87 Q8UHD7 Q8UHK4 Q8UI99
Q8UIE8 Q8UJ43 Q8VBT2 Q8VC19 Q8VEB6 Q8VR31 Q8VUR8 Q8VUS5 Q8VVC1 Q8W1X2
Q8X1S6 Q8X4V5 Q8X5X7 Q8X6X7 Q8X742 Q8X7E7 Q8X821 Q8X8N8 Q8X8T3 Q8X9Z9
Q8XA32 Q8XA44 Q8XAC5 Q8XBC7 Q8XBR7 Q8XCI7 Q8XCV5 Q8XCZ0 Q8XD09 Q8XD48
Q8XD57 Q8XD58 Q8XD99 Q8XDP0 Q8XDR6 Q8XE27 Q8XE76 Q8XEA7 Q8XEG2 Q8XEY8
Q8XGS1 Q8XHG1 Q8XHL8 Q8XI02 Q8XI46 Q8XIT0 Q8XJ22 Q8XJ27 Q8XJ28 Q8XJE1
Q8XJQ9 Q8XKN1 Q8XKU1 Q8XL65 Q8XMQ5 Q8XNC2 Q8XPB9 Q8XPE9 Q8XUU7 Q8XV80
Q8XW36 Q8XWT9 Q8XWY3 Q8XX95 Q8XXB6 Q8XXP9 Q8XXW5 Q8XXX5 Q8XYY7 Q8XZ29

Q8XZG4 Q8XZI7 Q8Y029 Q8Y052 Q8Y0A1 Q8Y0L5 Q8Y0Q3 Q8Y0U6 Q8Y0Y8 Q8Y249
Q8Y270 Q8Y2B9 Q8Y342 Q8Y3T4 Q8Y3X6 Q8Y4I3 Q8Y577 Q8Y5R8 Q8Y5S8 Q8Y5X8
Q8Y626 Q8Y663 Q8Y668 Q8Y6I2 Q8Y6J9 Q8Y6X2 Q8Y6X8 Q8Y708 Q8Y709 Q8Y7C1
Q8Y7G6 Q8Y822 Q8Y9N4 Q8YA20 Q8YAB8 Q8YAF2 Q8YB45 Q8YCV3 Q8YCX4 Q8YE79
Q8YEV0 Q8YFM2 Q8YGS6 Q8YH89 Q8YHF5 Q8YHH3 Q8YJK3 Q8YLZ0 Q8YM41 Q8YM73
Q8YMC2 Q8YMG7 Q8YNI3 Q8YP17 Q8YPC6 Q8YPW9 Q8YQ59 Q8YRL4 Q8YS26 Q8YT80
Q8YUA7 Q8YUB6 Q8YV89 Q8YXA2 Q8YXE4 Q8YZ80 Q8Z1U7 Q8Z268 Q8Z2H5 Q8Z2Q2
Q8Z2Y2 Q8Z308 Q8Z328 Q8Z3E0 Q8Z3X8 Q8Z3X9 Q8Z412 Q8Z4P4 Q8Z5C3 Q8Z5H2
Q8Z5J9 Q8Z5K0 Q8Z5N6 Q8Z5S9 Q8Z5W1 Q8Z699 Q8Z6I2 Q8Z787 Q8Z7L2 Q8Z7S9
Q8Z8B2 Q8Z8H8 Q8Z8S3 Q8Z8T4 Q8Z8X3 Q8Z9A5 Q8Z9B3 Q8Z9B4 Q8Z9I1 Q8Z9L3
Q8ZAQ5 Q8ZAX8 Q8ZBL9 Q8ZBM0 Q8ZBY7 Q8ZC45 Q8ZC94 Q8ZC96 Q8ZCA8 Q8ZCT6
Q8ZDW5 Q8ZE82 Q8ZES9 Q8ZEV0 Q8ZEY1 Q8ZF73 Q8ZFU4 Q8ZFX5 Q8ZFX6 Q8ZG01
Q8ZG91 Q8ZGA9 Q8ZGC4 Q8ZGY5 Q8ZH61 Q8ZHK5 Q8ZHV1 Q8ZIG9 Q8ZJF2 Q8ZJK9
Q8ZJP7 Q8ZKI4 Q8ZKL9 Q8ZKP7 Q8ZKX1 Q8ZLD7 Q8ZM82 Q8ZMA9 Q8ZNN4 Q8ZNT7
Q8ZNV2 Q8ZPS9 Q8ZQS2 Q8ZRA2 Q8ZRD1 Q8ZRP2 Q8ZRP8 Q8ZRX3 Q8ZTG3 Q8ZTJ7
Q8ZU54 Q8ZUG2 Q8ZZC7 Q90384 Q90703 Q90751 Q90752 Q90826 Q90997 Q90WJ8
Q90X23 Q90X24 Q91191 Q91309 Q91348 Q91636 Q91V12 Q91VM9 Q91XB0 Q91Y47
Q92037 Q92091 Q92209 Q922B2 Q922D8 Q922S4 Q92375 Q92403 Q924C3 Q925X9
Q926Z9 Q927D8 Q928B5 Q928I1 Q929H5 Q929I1 Q92A27 Q92A38 Q92A83 Q92AB2
Q92AD4 Q92AH1 Q92AH6 Q92AH7 Q92AV8 Q92AX5 Q92BF5 Q92BG1 Q92BJ3 Q92BJ4
Q92BN7 Q92BZ0 Q92C39 Q92CU0 Q92EG6 Q92EU4 Q92F47 Q92F90 Q92FZ0 Q92GA7
Q92GZ5 Q92HE8 Q92HI8 Q92HR1 Q92HY4 Q92I02 Q92I30 Q92IK8 Q92IX4 Q92J42
Q92J82 Q92JE7 Q92KY8 Q92L21 Q92MG0 Q92N62 Q92NH8 Q92NQ5 Q92Q22 Q92Q51
Q92QA1 Q92QB0 Q92QV4 Q92RJ1 Q92RR7 Q92SC6 Q92SC7 Q92SI9 Q92SK0 Q92SN8
Q92SX1 Q930J0 Q93112 Q93113 Q934J6 Q93AJ8 Q93AU4 Q93CH6 Q93GB7 Q93K97
Q93M42 Q93PS3 Q93R28 Q93T12 Q93UJ2 Q94655 Q94999 Q949X3 Q94IN5 Q95079
Q95081 Q95085 Q95086 Q95087 Q95088 Q95095 Q95182 Q95230 Q95334 Q95M18
Q95NM6 Q96282 Q96468 Q96GD0 Q96S42 Q96VL0 Q96WI0 Q96WL3 Q96WT9 Q96X43
Q96X44 Q970X0 Q970X1 Q970X5 Q973F1 Q976K2 Q977X5 Q979A8 Q97B58 Q97BG7
Q97C35 Q97CS3 Q97CT9 Q97E05 Q97E56 Q97EB7 Q97EE2 Q97ES6 Q97EV3 Q97EW5
Q97FJ7 Q97GK4 Q97GU0 Q97GU6 Q97HD5 Q97I62 Q97IQ6 Q97LD6 Q97LN7 Q97MR5
Q97MU2 Q97N11 Q97N17 Q97N21 Q97NC9 Q97ND6 Q97PI4 Q97PR0 Q97Q68 Q97RC8
Q97RS9 Q97RT8 Q97RW2 Q97S73 Q97SG0 Q97SR4 Q97Z88 Q980W4 Q980W5 Q983E9
Q985B1 Q985Y3 Q986N6 Q987C8 Q987T0 Q98AN7 Q98BM7 Q98C31 Q98DD5 Q98E57
Q98G10 Q98GG1 Q98GQ5 Q98HV0 Q98JB6 Q98KH9 Q98LC8 Q98LR2 Q98MB9 Q98ME7
Q98PF2 Q98PH6 Q98PH7 Q98PK9 Q98Q31 Q98QA8 Q98QG4 Q98QH1 Q98QM8 Q98QN9
Q98R67 Q98RF9 Q98RH5 Q99044 Q99046 Q99049 Q99055 Q99056 Q99172 Q99259
Q99376 Q99748 Q99988 Q99JF5 Q99JY8 Q99K85 Q99N42 Q99PS1 Q99SX3 Q99XH4
Q99XK9 Q99XL0 Q99XY1 Q99Y58 Q99YE2 Q9A076 Q9A077 Q9A0V7 Q9A115 Q9A178
Q9A2P3 Q9A5B6 Q9A671 Q9A6H0 Q9A6M5 Q9A707 Q9A733 Q9A734 Q9AAX8 Q9ABF9
Q9ABN3 Q9ABW5 Q9AC05 Q9AEN2 Q9AI36 Q9AM39 Q9ANR7 Q9AQ50 Q9AQC8 Q9BDW8
Q9BEA9 Q9BEB0 Q9BG99 Q9BGI0 Q9BGI3 Q9BQ50 Q9BQ52 Q9BQS2 Q9C0N4 Q9C2U0
Q9C401 Q9C4M5 Q9CB23 Q9CB28 Q9CBW0 Q9CCR4 Q9CDC1 Q9CDT1 Q9CE01 Q9CE78
Q9CE80 Q9CED2 Q9CEK9 Q9CEP1 Q9CEX2 Q9CF39 Q9CFC9 Q9CFJ0 Q9CFW9 Q9CGM7

Q9CGM8 Q9CGY9 Q9CHE0 Q9CHL8 Q9CHT5 Q9CHT8 Q9CII7 Q9CJ80 Q9CJD1 Q9CJL4
Q9CJN6 Q9CJW4 Q9CLE9 Q9CLM3 Q9CLT9 Q9CM20 Q9CMG7 Q9CMI7 Q9CMM1 Q9CN06
Q9CN86 Q9CNG9 Q9CNU1 Q9CNX8 Q9CNY5 Q9CPU0 Q9CQW5 Q9CZD3 Q9D142 Q9D819
Q9D964 Q9DAX2 Q9DBJ1 Q9ERS0 Q9EVE1 Q9EVG3 Q9EVG6 Q9EVG9 Q9EVH5 Q9EVH8
Q9EVI1 Q9EVI4 Q9EVI7 Q9EXQ1 Q9EYV3 Q9EYV5 Q9EYY6 Q9F1U7 Q9F1V2 Q9F2S0
Q9F323 Q9F6B2 Q9F7A2 Q9F7D4 Q9F8A8 Q9FB59 Q9FDN7 Q9FEW2 Q9FMT1 Q9FXM4
Q9FXM5 Q9GKM4 Q9GKR0 Q9GKX8 Q9GL72 Q9GL73 Q9GLD3 Q9GN94 Q9GTX8 Q9H2U2
Q9H777 Q9HB14 Q9HDQ1 Q9HDQ5 Q9HEY7 Q9HGY8 Q9HGY9 Q9HH76 Q9HHC2 Q9HIM0
Q9HIY5 Q9HJ19 Q9HJ84 Q9HKQ0 Q9HM15 Q9HMH9 Q9HN60 Q9HN82 Q9HNG2 Q9HRT1
Q9HRY0 Q9HSA4 Q9HUG8 Q9HV42 Q9HV51 Q9HVX0 Q9HVX6 Q9HVZ0 Q9HXJ5 Q9HXM6
Q9HXU0 Q9HXY2 Q9HYC7 Q9HZ68 Q9HZF8 Q9I099 Q9I0M6 Q9I3C5 Q9I3I0 Q9I4K6
Q9I6F1 Q9JI10 Q9JLT4 Q9JMH6 Q9JQM1 Q9JR00 Q9JRW9 Q9JT23 Q9JT57 Q9JT77
Q9JT95 Q9JTH8 Q9JTQ0 Q9JTR9 Q9JTS0 Q9JTT7 Q9JU79 Q9JUZ9 Q9JV18 Q9JVA3
Q9JVD6 Q9JW10 Q9JW13 Q9JW31 Q9JW59 Q9JW60 Q9JWP0 Q9JWQ5 Q9JX35 Q9JX66
Q9JXR2 Q9JXR3 Q9JXT8 Q9JXV7 Q9JXW0 Q9JY72 Q9JY95 Q9JYB4 Q9JYH7 Q9JYQ9
Q9JYT0 Q9JYT1 Q9JYU6 Q9JZI9 Q9JZX9 Q9K005 Q9K095 Q9K0D1 Q9K0U5 Q9K1D7
Q9K1G6 Q9K1Q0 Q9K3J3 Q9K4Z2 Q9K4Z7 Q9K715 Q9K7B5 Q9K866 Q9K8E9 Q9K8G3
Q9K8Y2 Q9K8Z3 Q9K971 Q9K9V7 Q9K9W2 Q9K9W3 Q9KA67 Q9KBZ6 Q9KC61 Q9KC78
Q9KCA8 Q9KCF1 Q9KD73 Q9KDG1 Q9KDG2 Q9KDH2 Q9KDV3 Q9KE51 Q9KEB0 Q9KF78
Q9KGG4 Q9KGK8 Q9KGN4 Q9KGU7 Q9KH06 Q9KJU4 Q9KL24 Q9KM62 Q9KMN7 Q9KNR1
Q9KNT5 Q9KNV7 Q9KP82 Q9KPV6 Q9KPY2 Q9KQI0 Q9KQT7 Q9KQW9 Q9KRZ2 Q9KS27
Q9KSF9 Q9KSS4 Q9KSU0 Q9KSX2 Q9KSZ6 Q9KT50 Q9KT52 Q9KT69 Q9KTC8 Q9KTL3
Q9KTW2 Q9KTX0 Q9KU60 Q9KU97 Q9KUT3 Q9KV27 Q9KVD5 Q9KWS8 Q9KXP2 Q9KXR3
Q9KXR7 Q9KYR6 Q9KZA7 Q9L0H2 Q9L278 Q9L2H4 Q9L2H9 Q9L3G6 Q9L516 Q9L6I2
Q9L7Z3 Q9L8F6 Q9L9I5 Q9LA24 Q9LCQ6 Q9LCT0 Q9LEL5 Q9LEL6 Q9M4S8 Q9M7M1
Q9MB95 Q9MT28 Q9MYU4 Q9MYV3 Q9MYY8 Q9MYZ3 Q9N0F3 Q9N1E2 Q9N1F5 Q9N2I7
Q9N2I8 Q9NAR7 Q9NG40 Q9NG42 Q9NL98 Q9NNW7 Q9NP81 Q9NZS2 Q9P3Y0 Q9P4T7
Q9P4V4 Q9P940 Q9PAE7 Q9PAF5 Q9PAR6 Q9PAX3 Q9PB04 Q9PB13 Q9PB43 Q9PB58
Q9PB95 Q9PBC2 Q9PBC6 Q9PCC5 Q9PCH2 Q9PE17 Q9PEB6 Q9PEE7 Q9PEH8 Q9PEQ0
Q9PFE2 Q9PFV8 Q9PG74 Q9PGJ7 Q9PGZ3 Q9PHB0 Q9PHM7 Q9PHZ3 Q9PIB3 Q9PIB4
Q9PIH8 Q9PII2 Q9PIR1 Q9PJF5 Q9PJF9 Q9PJJ9 Q9PJK0 Q9PJU2 Q9PK01 Q9PK18
Q9PK48 Q9PK58 Q9PK62 Q9PK66 Q9PKI3 Q9PKM3 Q9PKR5 Q9PKT7 Q9PKW1 Q9PKX8
Q9PLE1 Q9PLJ7 Q9PLW0 Q9PM34 Q9PMQ6 Q9PN49 Q9PNE6 Q9PP06 Q9PP70 Q9PP85
Q9PP99 Q9PPF4 Q9PPP3 Q9PPV6 Q9PPZ7 Q9PQ02 Q9PQC6 Q9PQK5 Q9PQK6 Q9PQW2
Q9PQW8 Q9PR38 Q9PR57 Q9PR71 Q9PR83 Q9PSM4 Q9PSN0 Q9PTQ2 Q9R008 Q9R0B9
Q9R0E1 Q9R0E2 Q9R1A9 Q9R229 Q9R6S7 Q9RA96 Q9RAM7 Q9RB09 Q9RBN6 Q9RDE4
Q9RDW6 Q9RFQ2 Q9RGW9 Q9RHV9 Q9RI00 Q9RN77 Q9RQ82 Q9RQ85 Q9RQ88 Q9RQF8
Q9RR97 Q9RRM7 Q9RRP1 Q9RSP3 Q9RSR5 Q9RT91 Q9RTH9 Q9RUB5 Q9RUN5 Q9RUN7
Q9RUP5 Q9RUV5 Q9RV62 Q9RVC3 Q9RWB2 Q9RWV7 Q9RWW0 Q9RX14 Q9RX68 Q9RXE1
Q9RXI8 Q9RXV7 Q9RY24 Q9RY51 Q9S3S1 Q9S3W0 Q9S5A5 Q9S5G6 Q9S6S1 Q9S795
Q9SA14 Q9SE93 Q9SKP6 Q9SMB8 Q9SML8 Q9SQL5 Q9SSY6 Q9TEM3 Q9TS45 Q9TSM4
Q9TSM5 Q9TUI8 Q9U4X2 Q9U4X3 Q9U4X4 Q9U4X5 Q9U8S9 Q9UDR5 Q9UHV8 Q9UKU6
Q9URB4 Q9URM2 Q9UVD6 Q9UWF8 Q9UX05 Q9UX09 Q9UX10 Q9UXV6 Q9UYR1 Q9UZ35
Q9UZP4 Q9V011 Q9V036 Q9V0L2 Q9V157 Q9V187 Q9V1I3 Q9V3P0 Q9VG94 Q9VG95

Q9VG96 Q9VG97 Q9VG98 Q9VNT5 Q9WTT6 Q9WV56 Q9WVK7 Q9WVL0 Q9WYG6 Q9WYG7
Q9WYW2 Q9WZ26 Q9WZF3 Q9WZJ9 Q9WZV4 Q9X0D0 Q9X0H5 Q9X0Y2 Q9X199 Q9X1A4
Q9X1B8 Q9X1F4 Q9X231 Q9X291 Q9X2E0 Q9X5F1 Q9X5V4 Q9X7B8 Q9X7W3 Q9X8R7
Q9X9S0 Q9XA06 Q9XAI3 Q9XBM7 Q9XC19 Q9XEA6 Q9XEA8 Q9XFW3 Q9XH57 Q9XH58
Q9XS49 Q9XS79 Q9XSX5 Q9XT75 Q9XZC0 Q9Y2T3 Q9Y617 Q9Y7F0 Q9Y7J0 Q9Y897
Q9Y8D9 Q9Y8G8 Q9Y9D8 Q9YAV8 Q9YAW4 Q9YBY3 Q9YFI5 Q9YHT4 Q9YIC2 Q9Z0J5
Q9Z0J6 Q9Z0J7 Q9Z0U5 Q9Z144 Q9Z1W4 Q9Z217 Q9Z2K8 Q9Z2K9 Q9Z339 Q9Z520
Q9Z5K9 Q9Z671 Q9Z6B9 Q9Z6H4 Q9Z6J6 Q9Z6J9 Q9Z6N1 Q9Z6X5 Q9Z736 Q9Z7A0
Q9Z7A4 Q9Z7P1 Q9Z7P2 Q9Z7Y7 Q9Z849 Q9Z851 Q9Z8I2 Q9Z8K3 Q9Z8M4 Q9Z901
Q9Z913 Q9Z9E2 Q9Z9F6 Q9ZB27 Q9ZBX1 Q9ZCA0 Q9ZCB9 Q9ZCG5 Q9ZCT4 Q9ZCZ4
Q9ZD32 Q9ZD55 Q9ZD76 Q9ZD97 Q9ZDA7 Q9ZDE7 Q9ZDL9 Q9ZDY3 Q9ZE17 Q9ZE56
Q9ZEJ7 Q9ZF99 Q9ZFC7 Q9ZHA7 Q9ZHE5 Q9ZHE6 Q9ZJD9 Q9ZJX0 Q9ZJX4 Q9ZK05
Q9ZK27 Q9ZK28 Q9ZKG4 Q9ZKG9 Q9ZKM5 Q9ZKZ1 Q9ZL18 Q9ZL69 Q9ZLL9 Q9ZLQ0
Q9ZLQ4 Q9ZLQ9 Q9ZM11 Q9ZM94 Q9ZMD0 Q9ZMJ6 Q9ZMM2 Q9ZMN8 Q9ZMP8 Q9ZMQ6
Q9ZMV3 Q9ZMW3 Q9ZMW6 Q9ZN29 Q9ZN53 Q9ZNC8 Q9ZP05 Q9ZP06 Q9ZVQ3 Q9ZWI9
Q9ZWL6

Swissprot Accession Number of 399 Homotrimers

O02765 O14684 O14788 O19072 O25927 O27367 O29912 O33507 O33980 O34275
O35235 O35734 O43557 O52503 O54693 O58398 O66862 O67089 O77510 O77764
O81821 O87792 O88310 O95150 O97605 O97626 P00480 P00481 P00501 P01375
P02931 P02932 P03275 P04501 P04924 P05099 P05430 P05452 P05654 P06804
P06968 P06996 P07206 P07219 P07238 P07996 P08011 P10440 P10562 P11066
P11724 P11725 P11803 P11818 P13296 P13415 P14267 P15141 P16303 P16599
P16883 P17579 P17876 P18047 P18048 P18194 P18195 P19080 P19101 P20148
P20149 P21420 P21941 P22121 P22335 P23383 P23510 P23563 P25006 P26972
P27547 P27548 P29553 P29715 P29965 P30297 P30687 P30688 P30689 P30690
P30691 P30692 P30704 P30705 P30952 P31326 P32018 P32199 P32200 P32201
P32970 P32971 P33620 P35441 P35442 P35448 P35773 P35774 P36711 P36845
P36846 P36847 P36848 P36939 P36940 P37432 P37592 P38501 P40852 P40853
P41008 P41047 P41151 P41152 P41273 P41354 P43025 P43488 P43839 P43859
P43887 P44584 P44815 P46025 P46027 P46542 P46544 P48023 P48094 P49077
P49714 P49744 P50477 P50591 P50592 P51435 P51742 P51743 P51749 P51942
P52278 P52705 P54080 P57042 P57339 P57440 P57454 P57494 P57708 P57762
P57765 P57954 P59659 P59684 P59693 P59694 P59695 P62617 P62618 P62619
P63304 P63305 P63306 P63307 P63308 P65183 P65184 P65320 P65321 P72215
P73426 P76335 P77747 P79066 P79337 P79374 P79382 P80094 P80463 P81445
P82142 P83079 P83129 P84010 P95379 P95781 P96134 P98095 Q00291 Q00740
Q01605 Q01606 Q02047 Q02420 Q02953 Q03350 Q04064 Q06006 Q06441 Q06599
Q06756 Q08016 Q12680 Q28178 Q40152 Q43064 Q43086 Q43087 Q43617 Q43814
Q46481 Q47490 Q47956 Q48216 Q48217 Q48218 Q48219 Q48220 Q48221 Q48473
Q51240 Q51813 Q52581 Q53239 Q54471 Q55746 Q56111 Q56113 Q56119 Q56828
Q57697 Q58502 Q60214 Q65914 Q65961 Q6FAU4 Q6HPT1 Q6LMT4 Q6NFC2 Q724H6

Q72HP8 Q72UP7 Q73FC0 Q73KC6 Q743W4 Q747A0 Q7MHQ5 Q7MXX0 Q7N8K6 Q7NC56
Q7NFH8 Q7NYL5 Q7UU80 Q7VZN1 Q7W5D0 Q7WCW4 Q81J62 Q81VV4 Q820F0 Q824F7
Q82GC9 Q82US7 Q839V8 Q87DY3 Q87EI4 Q87LQ3 Q87LZ1 Q87ME9 Q886L7 Q886N1
Q88MF3 Q88MG8 Q899E9 Q89AF8 Q89AN2 Q8A0Y7 Q8CVW1 Q8D224 Q8D2H3 Q8DBE9
Q8DBX0 Q8DC59 Q8DHC4 Q8EBR3 Q8F0A5 Q8FEJ6 Q8FMI4 Q8GB17 Q8HZD9 Q8JGW0
Q8K9D7 Q8K9H5 Q8K9I8 Q8K9R8 Q8KAZ0 Q8KC25 Q8MKG8 Q8NMB9 Q8P9Z0 Q8PAW5
Q8PLR7 Q8PML7 Q8PX25 Q8R6E7 Q8R7S8 Q8RFU2 Q8RQP5 Q8TUF7 Q8TWK3 Q8UFL3
Q8VCT4 Q8WNR1 Q8WWA0 Q8X8X8 Q8XE41 Q8XI08 Q8XYW2 Q8XZH9 Q8YAB4 Q8YQF0
Q8Z472 Q8Z9A2 Q8ZBP7 Q8ZH56 Q8ZMF7 Q8ZTY0 Q91VS7 Q92838 Q92F39 Q92JQ9
Q92Q45 Q92Z29 Q95116 Q95333 Q95MQ5 Q96320 Q96689 Q973F5 Q975N2 Q979S2
Q97LX0 Q97Z84 Q98MC6 Q99735 Q9A715 Q9BDM3 Q9BDM7 Q9BDN1 Q9BDN3 Q9BEA1
Q9BEA8 Q9BEG5 Q9CCW5 Q9CJK8 Q9HJQ0 Q9JM51 Q9JTM4 Q9JX26 Q9JYM5 Q9KGF7
Q9KPW4 Q9KUJ1 Q9L0Q7 Q9L4G1 Q9P9H8 Q9PDT5 Q9PEI5 Q9PIM1 Q9PJL1 Q9PJV8
Q9QYH9 Q9RXS6 Q9S7U5 Q9SCW4 Q9SCW5 Q9T0D3 Q9TLX4 Q9UWR9 Q9UYX8 Q9WZB5
Q9X6P4 Q9YFT8 Q9YHY9 Q9Z1T2 Q9Z2V2 Q9Z7Q4 Q9Z805 Q9ZED5 Q9ZJL7

Swissprot Accession Number of 1544 Homotetramers

O00097 O01360 O02691 O04997 O05118 O05969 O06134 O07165 O07400 O08309
O08325 O08333 O08756 O09452 O13276 O13277 O13278 O13309 O13507 O14556
O16027 O18866 O18867 O25067 O25116 O25657 O25836 O25883 O27090 O27120
O27441 O27527 O28323 O28542 O28965 O29195 O29515 O29627 O30853 O31046
O31287 O31616 O31776 O32755 O32765 O33734 O33832 O34154 O34268 O34425
O34529 O42259 O42615 O43026 O43175 O44006 O44104 O44105 O45687 O50316
O50965 O51114 O51323 O51522 O52605 O52631 O52866 O53446 O54804 O57479
O57672 O58033 O58498 O59494 O59536 O59841 O62619 O65175 O65198 O65199
O65595 O66271 O66605 O67161 O67216 O67353 O67862 O69177 O69294 O69300
O70351 O74101 O76243 O76463 O77695 O77834 O78310 O82845 O83327 O83536
O83816 O83971 O84348 O84366 O84863 O85063 O85070 O85347 O86840 O86841
O87761 O93344 O93401 O93537 O93538 O93539 O93540 O93541 O93542 O93543
O93544 O93545 O93546 O93619 O93620 O93868 O94038 O94122 O94788 O96423
O97524 O97764 P00305 P00330 P00331 P00332 P00335 P00336 P00337 P00338
P00339 P00340 P00341 P00342 P00343 P00344 P00345 P00352 P00354 P00355
P00356 P00357 P00358 P00359 P00360 P00361 P00362 P00365 P00492 P00493
P00494 P00496 P00497 P00511 P00512 P00548 P00549 P00562 P00722 P00805
P00927 P00935 P00944 P00950 P02339 P02776 P02777 P04034 P04076 P04181
P04182 P04406 P04422 P04424 P04642 P04788 P04796 P04797 P04968 P04970
P05034 P05042 P05091 P05640 P05707 P05792 P06106 P06148 P06150 P06151
P06169 P06219 P06220 P06608 P06614 P06622 P06672 P06721 P06758 P06760
P06765 P06801 P06977 P06981 P06995 P06998 P07003 P07195 P07343 P07346
P07439 P07486 P07487 P07505 P07754 P07833 P07846 P07864 P07913 P07999
P08074 P08127 P08177 P08236 P08294 P08328 P08417 P08439 P08477 P08735
P08843 P09033 P09124 P09151 P09316 P09317 P09380 P09381 P09383 P09464
P10096 P10097 P10172 P10173 P10182 P10528 P10537 P10618 P10654 P10655

P10792	P10889	P11155	P11415	P11550	P11603	P11716	P11884	P11954	P11964
P11974	P11979	P11980	P12070	P12265	P12268	P12269	P12310	P12382	P12628
P12762	P12804	P12851	P12928	P13029	P13242	P13490	P13491	P13601	P13697
P13714	P13715	P13743	P13798	P14178	P14295	P14408	P14561	P14618	P14673
P14674	P14675	P14831	P14940	P15034	P15115	P15157	P15437	P15587	P15723
P15875	P15944	P16115	P16125	P16243	P16467	P16468	P16551	P16858	P16932
P16960	P17244	P17262	P17329	P17330	P17331	P17445	P17721	P17729	P17730
P17750	P17819	P17878	P18022	P18157	P18310	P18757	P18840	P18949	P19079
P19089	P19148	P19205	P19314	P19315	P19629	P19808	P19858	P19869	P19871
P19992	P20000	P20035	P20231	P20275	P20369	P20373	P20445	P20506	P20619
P20673	P20839	P21164	P21170	P21599	P21777	P21778	P21817	P21867	P21938
P22200	P22221	P22281	P22303	P22360	P22512	P22513	P22842	P22857	P23234
P23368	P23631	P23722	P24163	P24164	P24165	P24166	P24299	P24300	P24547
P24707	P24746	P24748	P24749	P24750	P24751	P24753	P25524	P25529	P25553
P25716	P25762	P25795	P25858	P25861	P25984	P26263	P26283	P26517	P26518
P26519	P26520	P26521	P26616	P26899	P26900	P26988	P26997	P27138	P27157
P27346	P27435	P27443	P27463	P27605	P27725	P27726	P27756	P27887	P28032
P28042	P28043	P28044	P28045	P28046	P28173	P28227	P28248	P28577	P28578
P28581	P28595	P28643	P28844	P28894	P28910	P29132	P29272	P29441	P29442
P29443	P29497	P29758	P29993	P30034	P30035	P30146	P30348	P30363	P30435
P30613	P30614	P30615	P30616	P30724	P30754	P30835	P30837	P30957	P31212
P31373	P31865	P32044	P32169	P32320	P32427	P32635	P32636	P32637	P32638
P32809	P32810	P32929	P33109	P33149	P33158	P33207	P33380	P33571	P33572
P33898	P34038	P34105	P34734	P34783	P34916	P34917	P34918	P34919	P34920
P34921	P34922	P34923	P34924	P35143	P35497	P35520	P35523	P36214	P36444
P36624	P36774	P36959	P37020	P37031	P37112	P37136	P37222	P37223	P37435
P39460	P39461	P39482	P39483	P39484	P39485	P40111	P40288	P40369	P40733
P40881	P40927	P40947	P41261	P41262	P41747	P41906	P41975	P42119	P42120
P42121	P42122	P42123	P42329	P42455	P42556	P42829	P42908	P43067	P43279
P43503	P43577	P43797	P43861	P43863	P43864	P43924	P44304	P44324	P44334
P44341	P44398	P44409	P44502	P44505	P44539	P44881	P45105	P45382	P45395
P45396	P45600	P45687	P46067	P46154	P46406	P46493	P46533	P46547	P46562
P46713	P46795	P46796	P46807	P46863	P47191	P47458	P47481	P47543	P47698
P47738	P47856	P47857	P47858	P47959	P48026	P48163	P48571	P48575	P48576
P48644	P48812	P49189	P49383	P49384	P49385	P49419	P49423	P49431	P49433
P49644	P49724	P50096	P50097	P50200	P50286	P50321	P50322	P50388	P50455
P50514	P50910	P50933	P50934	P51009	P51181	P51182	P51464	P51469	P51615
P51640	P51647	P51649	P51650	P51844	P51977	P52200	P52476	P52480	P52489
P52694	P52700	P52713	P52977	P52987	P53429	P53430	P53607	P53657	P54117
P54118	P54202	P54226	P54270	P54272	P54273	P54622	P55070	P55071	P55971
P55995	P56069	P56088	P56149	P56389	P56511	P56512	P56533	P56649	P56681
P56744	P57197	P57290	P57300	P57384	P57404	P57491	P57549	P57610	P58072
P58156	P58207	P58554	P58559	P58837	P58838	P58898	P58899	P58900	P58901
P59039	P59040	P59050	P59189	P59390	P59407	P59409	P59410	P59443	P59577

P60560 P60561 P61879 P61880 P62002 P62003 P63476 P63477 P63901 P63902
P63943 P63944 P63945 P63946 P63947 P63948 P63949 P64172 P64173 P64178
P64179 P64180 P64181 P64413 P64414 P65168 P65255 P65256 P65257 P65258
P65259 P65260 P65261 P65692 P65693 P65921 P65922 P65923 P65924 P65925
P65926 P66875 P66876 P66930 P66931 P67653 P67654 P68776 P68777 P68838
P68839 P70685 P70789 P71384 P71711 P72156 P72220 P73053 P74208 P74762
P74956 P75358 P77810 P77832 P77983 P78007 P78025 P78031 P78958 P78986
P79912 P79913 P80019 P80038 P80039 P80064 P80092 P80447 P80505 P80506
P80534 P80645 P80869 P80885 P81006 P81178 P83401 P87025 P87197 P87208
P93031 P93052 P93407 P94565 P94685 P94907 P94915 P94939 P95331 P95477
P96351 P96420 P97807 P99067 P99072 P99119 P99123 Q00301 Q00584 Q00630
Q00640 Q00796 Q01077 Q01134 Q01360 Q01462 Q01558 Q01597 Q01612 Q01651
Q01782 Q01982 Q02141 Q02145 Q02252 Q02253 Q02338 Q02499 Q02912 Q03720
Q04285 Q04513 Q04520 Q04657 Q04668 Q04796 Q04837 Q05025 Q06004 Q06176
Q06178 Q06203 Q07234 Q07288 Q07636 Q07637 Q07786 Q07805 Q07841 Q08257
Q08460 Q09054 Q09737 Q10208 Q12552 Q12629 Q12634 Q12669 Q14571 Q15413
Q15661 Q17334 Q27652 Q27686 Q27743 Q27788 Q27797 Q27888 Q27890 Q27895
Q28204 Q28259 Q28265 Q28399 Q28452 Q28554 Q28999 Q29228 Q29318 Q29490
Q29491 Q29499 Q29529 Q29536 Q29558 Q29563 Q39734 Q39735 Q39769 Q40546
Q41247 Q41595 Q42671 Q42806 Q42910 Q42954 Q42977 Q43247 Q44185 Q44473
Q44524 Q45515 Q46078 Q46289 Q46381 Q46450 Q48335 Q48965 Q49729 Q51404
Q52087 Q52998 Q54775 Q55513 Q55674 Q55863 Q56216 Q56301 Q56581 Q57572
Q57603 Q57665 Q57969 Q58130 Q58133 Q58546 Q58819 Q59007 Q59092 Q59126
Q59185 Q59199 Q59202 Q59214 Q59244 Q59309 Q59321 Q59435 Q59545 Q59645
Q59800 Q59827 Q59828 Q59906 Q59925 Q60009 Q60022 Q60176 Q61753 Q62148
Q62976 Q63269 Q63639 Q64057 Q64442 Q64531 Q64565 Q64591 Q6FIS9 Q6FPW3
Q6FSM4 Q6FV12 Q6LY59 Q703I2 Q74MP4 Q757I2 Q759A9 Q7A9X4 Q7C637 Q7M9W4
Q7MD57 Q7MP77 Q7N129 Q7N4H8 Q7N4P7 Q7NG49 Q7NI93 Q7NZ02 Q7P0H2 Q7RVA8
Q7SX99 Q7TUV5 Q7U0N6 Q7U892 Q7U8I1 Q7UI51 Q7UVG2 Q7V024 Q7V5R2 Q7VA47
Q7VBG1 Q7VH30 Q7VKC9 Q7VQJ6 Q7VR50 Q7W0A2 Q7W4N9 Q7WG65 Q7WYP6 Q7X5C9
Q7Z4W1 Q814T2 Q815X8 Q816G3 Q81EP4 Q81F85 Q81JW1 Q81K80 Q81RW4 Q81SA0
Q81T68 Q81XJ7 Q820M0 Q822D5 Q822T2 Q82BV3 Q82ID7 Q82SM5 Q838C9 Q838L1
Q839C1 Q83CL8 Q83JF4 Q83ML8 Q83PE1 Q83SM9 Q83SP0 Q867C9 Q875M9 Q875S4
Q875Z9 Q87AT3 Q87CL8 Q87DY8 Q87H06 Q87LP9 Q87SS7 Q880Z4 Q885V0 Q886M5
Q886Y1 Q88K39 Q88M20 Q88MG1 Q88P28 Q88Z76 Q890M6 Q892U0 Q89A53 Q89A99
Q89AI8 Q89AK1 Q89AR4 Q89AY0 Q89GB0 Q89VC7 Q89XM2 Q8A1A0 Q8A9M2 Q8AXL1
Q8AYS8 Q8BWF0 Q8CMZ0 Q8CNI2 Q8CNL3 Q8CNR1 Q8CNY0 Q8CP96 Q8CPY5 Q8CUH5
Q8D1Q8 Q8D2K0 Q8D2L3 Q8D2M3 Q8D442 Q8D632 Q8DBB0 Q8DC63 Q8DEE1 Q8DIP7
Q8DJ32 Q8DJK4 Q8DKT7 Q8DUE5 Q8DZY3 Q8E5N4 Q8E8J1 Q8E9N2 Q8EFT7 Q8ELF0
Q8ELU7 Q8EM53 Q8EN67 Q8EQJ1 Q8ESW9 Q8EUG3 Q8F132 Q8F445 Q8F9L0 Q8FBD0
Q8FBE1 Q8FCA2 Q8FCE3 Q8FD58 Q8FD73 Q8FDU7 Q8FE34 Q8FHA7 Q8FJ93 Q8FL13
Q8FL75 Q8FLV6 Q8FP04 Q8FQP8 Q8FU05 Q8FX90 Q8FZC4 Q8G0G1 Q8G0I7 Q8G1R0
Q8G204 Q8G3Q1 Q8G527 Q8GMJ0 Q8HXZ7 Q8HXZ8 Q8HY00 Q8HY01 Q8HY02 Q8HY03
Q8HY04 Q8HY06 Q8HY10 Q8HY11 Q8HY12 Q8HYC0 Q8HYE4 Q8J1H3 Q8K5G1 Q8K8M9

Q8K933 Q8K988 Q8K9M3 Q8K9U0 Q8K9U9 Q8KA24 Q8KC06 Q8KCW4 Q8KN28 Q8KQC4
Q8KTE1 Q8L0Z4 Q8MI17 Q8NK47 Q8NKN9 Q8NLN0 Q8NQL7 Q8NRN8 Q8NUM9 Q8NVV1
Q8NWS5 Q8NYC7 Q8NZF8 Q8P3H1 Q8P9T9 Q8P9V6 Q8P9Z6 Q8PEW5 Q8PLL9 Q8PLN5
Q8PLS3 Q8PNN2 Q8PTD3 Q8PXE2 Q8R146 Q8R720 Q8R7K0 Q8RBI5 Q8RCF9 Q8RDK3
Q8RDN6 Q8RED8 Q8RL85 Q8RQM8 Q8RQM9 Q8SPU8 Q8TFJ2 Q8TGZ7 Q8THB0 Q8TS07
Q8TUT9 Q8TWE6 Q8TXF9 Q8TZV1 Q8U3C9 Q8U429 Q8U7G6 Q8U8I2 Q8UD63 Q8UEY5
Q8UEY7 Q8UGL3 Q8VCN5 Q8VID1 Q8WNV7 Q8WYP3 Q8WZN0 Q8X1X3 Q8X769 Q8X8A1
Q8X8Y9 Q8X984 Q8X9J0 Q8X9Z8 Q8XCX9 Q8XDD9 Q8XEJ1 Q8XHQ7 Q8XIB3 Q8XJ56
Q8XP62 Q8XQE8 Q8XSZ5 Q8XXP1 Q8Y099 Q8Y0B8 Q8Y3I8 Q8Y495 Q8Y551 Q8Y5R9
Q8Y6Z6 Q8Y766 Q8YB50 Q8YCP2 Q8YFX5 Q8YG60 Q8YHC6 Q8YHF2 Q8YIJ3 Q8YMD4
Q8YQY1 Q8Z2F4 Q8Z303 Q8Z3F0 Q8Z3G5 Q8Z3G6 Q8Z4R8 Q8Z6K2 Q8Z6R6 Q8Z9B1
Q8Z9F9 Q8Z9I0 Q8Z9Z1 Q8ZB47 Q8ZBI2 Q8ZBM5 Q8ZBN1 Q8ZCD0 Q8ZEB6 Q8ZIG8
Q8ZJ03 Q8ZJN2 Q8ZKS1 Q8ZL52 Q8ZL90 Q8ZLQ6 Q8ZLS0 Q8ZLS1 Q8ZN71 Q8ZPL7
Q8ZRT5 Q8ZVI1 Q8ZVL3 Q8ZWD6 Q8ZWK7 Q8ZX28 Q90ZC7 Q91X52 Q91XV4 Q91YI0
Q92055 Q920N9 Q920P0 Q92122 Q92211 Q92243 Q92263 Q92345 Q92441 Q926R2
Q92736 Q927T4 Q929E8 Q92A28 Q92BI0 Q92BS0 Q92F65 Q92GW0 Q92I25 Q92I97
Q92IL5 Q92LW9 Q92PB6 Q92QA0 Q92R55 Q92WP0 Q939R0 Q93DW6 Q93HF3 Q93RJ9
Q94469 Q95028 Q95J96 Q95LC6 Q95V25 Q96UF1 Q96UF2 Q96US8 Q96YF6 Q971K2
Q971T8 Q974Y8 Q975R3 Q976J7 Q979S5 Q97BJ8 Q97D80 Q97DC6 Q97F61 Q97GI9
Q97MC5 Q97MD1 Q97R25 Q97S93 Q97VM8 Q97ZF4 Q97ZY3 Q980H6 Q983J7 Q983U5
Q98A07 Q98CR8 Q98HN3 Q98MF0 Q98PG4 Q98PQ3 Q98SK9 Q98SL0 Q98SL1 Q98SL2
Q99714 Q99KE1 Q99LB2 Q9A6I5 Q9A7K3 Q9A823 Q9A900 Q9AGC0 Q9AKE4 Q9AKJ9
Q9AKQ3 Q9AL83 Q9APM5 Q9BE24 Q9BG98 Q9BTZ2 Q9BZJ3 Q9C136 Q9C4Y9 Q9CB76
Q9CBW4 Q9CF61 Q9CFG7 Q9CG73 Q9CGG8 Q9CI75 Q9CII4 Q9CJN5 Q9CJW9 Q9CKB0
Q9CKJ2 Q9CLZ7 Q9CMK1 Q9DBF1 Q9DCZ1 Q9EVE3 Q9EVG4 Q9EVH0 Q9EVH6 Q9EVI8
Q9EVR0 Q9EZ12 Q9FBN6 Q9GKX2 Q9H2X3 Q9HFX1 Q9HGY7 Q9HJ69 Q9HJQ5 Q9HLB6
Q9HQ29 Q9HQ52 Q9HSS7 Q9HXK5 Q9HXZ4 Q9I407 Q9I4W3 Q9I587 Q9JHW6 Q9JTK1
Q9JTR0 Q9JUK6 Q9JUU9 Q9JYJ8 Q9JZG1 Q9JZR4 Q9K5Z8 Q9K6D7 Q9K843 Q9K8E8
Q9K993 Q9KA91 Q9KC32 Q9KCX4 Q9KGU2 Q9KHR3 Q9KL62 Q9KMW9 Q9KP83 Q9KPC4
Q9KQ47 Q9KWN1 Q9L0B8 Q9L233 Q9L558 Q9L6B7 Q9N2D1 Q9N655 Q9NNX6 Q9P4B5
Q9P4B6 Q9P4C2 Q9P6C8 Q9P7P7 Q9P8C0 Q9PCG3 Q9PDU1 Q9PER5 Q9PJ84 Q9PJ85
Q9PJN6 Q9PK33 Q9PK50 Q9PK61 Q9PKL0 Q9PL64 Q9PLV9 Q9PPB4 Q9PQK7 Q9PRH8
Q9PT42 Q9PT43 Q9PVK4 Q9PVK5 Q9PW04 Q9PW05 Q9PW06 Q9PW07 Q9PW58 Q9PW61
Q9RH76 Q9RR70 Q9RTU4 Q9RU23 Q9RUA9 Q9S224 Q9S306 Q9S3Z4 Q9S4K9 Q9SNY3
Q9SYG7 Q9TSX5 Q9UJ83 Q9UR38 Q9UVC0 Q9UW96 Q9UWN5 Q9UXB2 Q9UXX2 Q9UY33
Q9UZ51 Q9V1N3 Q9V1P1 Q9VW26 Q9W719 Q9W7K5 Q9W7L3 Q9W7L4 Q9W7L5 Q9W7M6
Q9WYT0 Q9WZ23 Q9WZR0 Q9X1K9 Q9X1Z5 Q9X7L2 Q9X9W0 Q9XSM2 Q9XT86 Q9XT87
Q9Y3B8 Q9Y796 Q9Y8E9 Q9YAW1 Q9YBF1 Q9YFS9 Q9YGL2 Q9YI05 Q9Z126 Q9Z244
Q9Z518 Q9Z5C5 Q9Z6K9 Q9Z6P6 Q9Z7T0 Q9Z8U8 Q9Z984 Q9Z9F4 Q9Z9U1 Q9ZCQ4
Q9ZD92 Q9ZDF1 Q9ZDM6 Q9ZEY8 Q9ZJ93 Q9ZJL3 Q9ZJQ9 Q9ZKT0 Q9ZL14 Q9ZLI5
Q9ZM13 Q9ZM99 Q9ZMW7 Q9ZPB7

Swissprot Accession Number of 38 Homopentamers

O33421 P16328 P24216 P35443 P39738 P42274 P45048 P58297 P67910 P67911
P67912 P67913 Q51061 Q72ET7 Q7MPN6 Q7NTL6 Q7VKK8 Q7VZF5 Q7W609 Q7WGU9
Q83PP2 Q87T56 Q8DE09 Q8FCA0 Q8RIA5 Q8Y0X8 Q8ZJN4 Q98I52 Q9CL97 Q9HYQ8
Q9JQX8 Q9K002 Q9K3C5 Q9KQ63 Q9KWW7 Q9PHW6 Q9X0K7 Q9ZL91

Swissprot Accession Number of 277 Homohexamers

O08444 O13465 O26010 O26824 O28303 O32810 O33808 O34797 O35459 O51645
O51891 O52250 O52310 O57940 O59650 O66143 O66614 O67031 O68883 O69466
O74024 O83281 O83307 O83716 O83990 O93934 P00366 P00367 P00368 P00369
P00370 P00510 P00814 P00891 P03002 P03692 P07262 P08205 P08298 P08955
P09743 P09948 P10860 P13154 P14165 P14193 P14604 P14657 P15111 P15168
P17288 P17346 P17556 P17557 P18789 P18819 P20054 P20901 P20902 P20960
P21161 P23875 P24295 P26443 P26491 P26980 P27660 P27708 P28302 P28724
P28997 P29051 P29464 P29507 P30084 P31026 P32321 P32382 P33561 P34559
P38480 P38527 P38576 P39475 P39708 P42457 P43387 P43770 P43793 P44417
P44619 P44805 P45616 P45835 P47232 P47233 P47295 P47724 P49448 P50308
P50389 P50870 P51042 P52152 P52153 P52154 P52155 P52156 P54386 P54387
P54388 P54531 P55990 P56463 P56466 P57606 P57643 P57652 P58103 P58228
P59099 P59293 P59578 P63814 P63815 P63816 P63817 P63818 P63819 P63820
P63821 P63822 P63823 P65933 P65934 P66028 P66029 P71154 P75053 P78804
P80053 P80063 P80319 P82264 P94316 P94598 P95544 P96110 Q01468 Q03222
Q03432 Q03578 Q05650 Q06447 Q08352 Q10530 Q13825 Q47950 Q47951 Q48296
Q50452 Q53560 Q55235 Q55435 Q56037 Q56304 Q57961 Q58576 Q59194 Q59482
Q62651 Q6GA90 Q6GHW1 Q87EM8 Q87T80 Q88AH3 Q88CQ7 Q88VC8 Q895N8 Q89A22
Q89A58 Q89L55 Q8A3C0 Q8CSZ5 Q8CX61 Q8D2R5 Q8DDY6 Q8DJQ7 Q8DNE6 Q8DVH2
Q8E1A6 Q8E6R1 Q8E8I0 Q8ER64 Q8EYP6 Q8FC88 Q8FPP9 Q8G7H5 Q8K937 Q8KDS9
Q8KRR5 Q8NQU5 Q8P857 Q8PJK5 Q8PY58 Q8R9U9 Q8RGX1 Q8RQD2 Q8RQP4 Q8RSX4
Q8RT67 Q8TFF6 Q8TI88 Q8TX03 Q8U4H3 Q8UES4 Q8VW75 Q8XJM7 Q8Y2E6 Q8Y5K7
Q8Z2H1 Q8Z421 Q8Z6F6 Q8ZH64 Q8ZH86 Q8ZJN9 Q8ZL48 Q8ZMB8 Q929W5 Q92PY8
Q93JW0 Q96T66 Q96UJ9 Q96VJ7 Q975U2 Q97IB2 Q97NQ2 Q980M1 Q98M51 Q98RB3
Q99JR6 Q9CDQ6 Q9CH10 Q9CLD4 Q9HFR6 Q9HGU3 Q9HGU4 Q9HNP9 Q9HRW6 Q9HSM4
Q9I6D1 Q9JLZ3 Q9K9Q6 Q9KVC4 Q9PEP8 Q9PPF2 Q9RHM8 Q9RME4 Q9RWM4 Q9S1F9
Q9URS1 Q9V287 Q9WZK0 Q9X980 Q9XC89 Q9YAC7 Q9YC65 Q9Z431 Q9Z613 Q9ZBR1
Q9ZD24 Q9ZEU7 Q9ZI54 Q9ZJE4 Q9ZK38 Q9ZKD8 Q9ZLS9

Swissprot Accession Number of 116 Homooctamers

O00088 O04867 O22504 O22506 O30808 O33946 O33949 O35156 O82560 O95749
P00965 P04078 P04770 P04771 P04772 P04773 P04841 P07694 P08281 P08282
P08310 P08695 P09606 P10887 P11122 P11444 P11600 P12033 P12424 P13453
P14636 P14654 P14655 P14656 P15047 P15102 P15103 P15104 P15105 P16580
P17297 P19432 P19923 P19924 P20477 P20478 P20805 P22073 P22878 P23015
P23712 P24099 P25462 P26914 P28316 P28516 P32288 P32289 P34497 P38559

P38560 P38561 P38562 P38563 P41320 P45513 P45626 P46410 P51118 P51119
P51121 P51845 P51846 P51847 P51848 P51849 P51850 P51851 P52782 P52783
P56966 P79303 Q00922 Q02154 Q04831 Q06378 Q07130 Q07131 Q09179 Q12613
Q42624 Q42688 Q42689 Q42899 Q43066 Q43127 Q43785 Q43931 Q59477 Q60030
Q6FMT6 Q75BT9 Q86ZF9 Q86ZU6 Q874T6 Q8HZM5 Q8J1R3 Q8X169 Q96UG9 Q96UV5
Q96V52 Q9C2U9 Q9LVI8 Q9UUN6 Q9WTN0 Q9XQ94
